# A Copper-Selective Sensor and Its Inhibition of Copper-Amyloid Beta Aggregation

**DOI:** 10.3390/bios14050247

**Published:** 2024-05-14

**Authors:** Ngoc Kim Nguyen, Bella Poduska, Mia Franks, Manoranjan Bera, Ian MacCormack, Guoxing Lin, Alexander P. Petroff, Samir Das, Arundhati Nag

**Affiliations:** Carlson School of Chemistry and Biochemistry, Clark University, 950 Main Street, Worcester, MA 01610, USA; lekimberly99@gmail.com (N.K.N.); bpoduska@clarku.edu (B.P.); mgfranks@usc.edu (M.F.); mbera@clarku.edu (M.B.); imaccormack@clarku.edu (I.M.); glin@clarku.edu (G.L.); apetroff@clarku.edu (A.P.P.); samdas@clarku.edu (S.D.)

**Keywords:** Cu-amyloid beta, non-amyloid aggregate inhibition, fluorescent copper chelator

## Abstract

Copper is an essential trace metal for biological processes in humans and animals. A low level of copper detection at physiological pH using fluorescent probes is very important for in vitro applications, such as the detection of copper in water or urine, and in vivo applications, such as tracking the dynamic copper concentrations inside cells. Copper homeostasis is disrupted in neurological diseases like Alzheimer’s disease, and copper forms aggregates with amyloid beta (Ab42) peptide, resulting in senile plaques in Alzheimer’s brains. Therefore, a selective copper detector probe that can detect amyloid beta peptide-copper aggregates and decrease the aggregate size has potential uses in medicine. We have developed a series of Cu^2+^-selective low fluorescent to high fluorescent tri and tetradentate dentate ligands and conjugated them with a peptide ligand to amyloid-beta binding peptide to increase the solubility of the compounds and make the resultant compounds bind to Cu^2+^–amyloid aggregates. The copper selective compounds were developed using chemical scaffolds known to have high affinity and selectivity for Cu^2+^, and their conjugates with peptides were tested for affinity and selectivity towards Cu^2+^. The test results were used to inform further improvement of the next compound. The final Cu^2+^ chelator–peptide conjugate we developed showed high selectivity for Cu^2+^ and high fluorescence properties. The compound bound 1:1 to Cu^2+^ ion, as determined from its Job’s plot. Fluorescence of the ligand could be detected at nanomolar concentrations. The effect of this ligand on controlling Cu^2+^–Ab42 aggregation was studied using fluorescence assays and microscopy. It was found that the Cu^2+^–chelator–peptide conjugate efficiently reduced aggregate size and, therefore, acted as an inhibitor of Ab42-Cu^2+^ aggregation. Since high micromolar concentrations of Cu^2+^ are present in senile plaques, and Cu^2+^ accelerates the formation of toxic soluble aggregates of Ab42, which are precursors of insoluble plaques, the developed hybrid molecule can potentially serve as a therapeutic for Alzheimer’s disease.

## 1. Introduction

Copper is an essential element in organisms [1,2] and many cytosolic [3], mitochondrial [4], and vesicular [5] enzymes require copper ion (Cu^2+^ or Cu^1+^) as a cofactor for their activities [6]. Therefore, the detection of copper ions has been a significant topic of research, both in vitro, such as the detection of Cu^2+^ in water [7] or urine [8], and in vivo, such as tracking the dynamic copper ion movement inside cells [9]. Fluorescent sensors [10,11,12] have been widely used for detecting and monitoring Cu^2+^ due to their high biocompatibility [13]. Copper has also been recently detected by peptide sensors such as the dipeptide Trp-Phe [14], the human Gly-His-Lys peptide [15], and peptide sensors discovered through peptide library screening such as Ser-Ala-Gln-Ile-Ala-Pro-His [16].

Copper homeostasis is essential for maintaining normal brain functions [17], and dysregulation of copper homeostasis is seen in neurodegenerative diseases such as Alzheimer’s disease (AD) [18]. Cu^2+^ is found in high concentrations in senile plaques, which comprise amyloid-β (Ab) peptides aggregated to form β-sheet rich fibrils. Ab peptides are generated from the amyloidogenic cleavage pathway of amyloid precursor protein (APP), due to sequential action of membrane-bound β- and γ-secretases on APP. The β-secretase cleaves the APP extracellular domain, generating a membrane-tethered β-C-terminal fragment of APP [19]. This transmembrane β-C-domain is cleaved by γ-secretase to generate the Ab peptides ranging in length from 38 to 43 residues [20]. The Ab42 variant is more hydrophobic and prone to fibril formation than Aβ40. Cu^2+^ can bind Ab and thus modulate the aggregation process [21]. Ab bound to redox-active metal ions such as Cu^2+^ are considered more toxic since they can produce reactive oxygen species (ROS), damaging the surrounding biomolecules [22]. Metals such as Zn^2+^ and Cu^2+^ form salt bridges predominantly through a His13-metal-His14 coordination and bridge with His6 [23]. Cu^2+^ has been shown to bind to His with greater affinity than Zn^2+^ and significantly stabilizes Ab aggregates [24]. The reduction of Cu^2+^–amyloid complexes to Cu^1+^–amyloid complexes produces hydrogen peroxide, leading to the synthesis of pro-apoptotic lipid peroxidation products, which, in turn, induces neuronal cell death in AD [25]. Therefore, designing bifunctional ligands that can inhibit Ab aggregation and target metal homeostasis is an attractive strategy that has been explored [26,27]. However, the reported ligands do not have intrinsic fluorescent properties or selectivity for Cu^2+^ and adding a fluorescent dye to a small ligand can significantly change its character and how it interacts with biomolecules. Here, we report on developing a fluorescent Cu^2+^-selective bifunctional ligand that could bind to amyloid beta aggregates and detect Cu^2+^. The bifunctional ligand consisted of two parts—the first part being a known Ab42 peptide binder that prevented amyloid aggregation and the second part being a ligand framework that selectively chelated to Cu^2+^ ions.

Ab42 contains two hydrophobic regions, Ala30–Val36 (at the C terminal) and Leu17–Ala21, referred to as the Central Hydrophobic Core (CHC) [28,29]. Ab-based peptide inhibitors are based on the structure of the C-terminal fragments (CTF) and the CHC sequences of the Ab peptide [30]. They bind to the Ab peptide at specific sites and prevent its assembly into amyloid fibrils. ^17^ LVFAA^21^, the CHC sequence of Ab42 is self-recognizing and a critical nucleation site, and therefore, peptide inhibitors have been derived to prevent the self-recognition and resultant nucleation at this site. The hydrophobic peptide LPFFD was derived by replacing valine with proline and alanine with aspartic acid in ^17^LVFFA^21^ [31,32]. It recognized the CHC of the Ab fibril and inhibited Ab fibril aggregation. LPFFD also reduced plaque load and decreased neurotoxicity [32]. LPFFD contains proline, a beta-sheet breaker, and the lack of a proton on the secondary substituted nitrogen in the peptide bond of proline residue could inhibit the formation of the intramolecular hydrogen bonds into fibrils [32]. Therefore, we chose LPFFD as the inhibitory peptide arm or the first part of our bifunctional ligand.

Next, we focused on developing tridentate or tetradentate imidazole or thiazole-based fluorescent cores, focusing on Cu^2+^ selectivity. We synthesized a previously reported tetradentate core, A1, and designed and synthesized a new tetradentate core (A2) and a new thiazole-based core (A3) to coordinate Cu^2+^. The tridentate or tetradentate imidazole and thiazole-based ligands were not soluble in the buffer. When the tridentate or tetradentate ligand was coordinated with the peptide, the resultant peptide–chelator hybrid compounds became water soluble, allowing Cu^2+^ detection in an aqueous condition at a physiologically relevant pH. Moreover, the final compound, L3, that contained the A3 metal-chelating core was selective for Cu^2+^ over physiological concentrations of other metal ions. Finally, we demonstrated that this peptide–chelator hybrid compound decreased the aggregation of Ab-Cu^2+^ aggregates using fluorescence assays and microscopy.

## 2. Materials and Methods

### 2.1. Materials

All materials used were purchased from Sigma Aldrich unless stated otherwise. Ascorbic acid and 2,6-lutidine were purchased from Alfa Aesar. DMF (N, N′-dimethylformamide) and DCM (dichloromethane) and NMP (1-methyl-2-pyrrolidinone and DIEA (N, N-diisopropylethylamine) were purchased from VWR, ChemPep, and Chem-Impex, respectively. Acetonitrile (ACN) was purchased from VWR. All amino acids used in the peptide synthesis were purchased from Chem-Impex and ChemPep. Rink amide resin (loading 0.51 mmol/g, size 100–200 mesh and HATU ((2-(7-Aza-1H-benzotriazole-1-yl)- 1,1,3,3-tetramethylammoniumhexafluorophosphate) were purchased from ChemPep. Coupling reagents used in automated peptide synthesis, such as Oxyma (Ethyl cyanohydroxyiminoacetate), DIC (N, N′-Diisopropylcarbodiimide), and HOBt (Hydroxybenzotriazole) were purchased from Chem-Impex. Copper (II) acetate, iron(III) nitrate, manganese (II) acetate, and nickel (II) sulfate were purchased from Sigma Aldrich. Sodium sulfate, zinc (II) nitrate, and cobalt (II) sulfate were purchased from Fisher Scientific. Magnesium (II) sulfate was purchased from Amreco. The fluorescence assays were carried out in buffer solutions of HEPES (Acros). Amyloid beta 1-42 (Ab42) was purchased from GeneScript. The peptide was dissolved in HFIP (hexafluoroisopropanol) purchased from TCI America. ThT (Thioflavin T) was purchased from Thermo Scientific (Waltham, MA, USA).

### 2.2. Synthesis of Metal Chelator A1

A1 was synthesized by modifying the literature procedure [33]. A total of 23.9 mmol of N-Methyl-1,2-phenylenediamine dihydrochloride was reacted with 11.4 mmol of iminodiacetonitrile in a reflux reaction for 100 h with 3 M HCl solvent to form the metal chelator A1. The product was washed with ammonium hydroxide to form a precipitate. The precipitate was recrystallized. Following the reflux reaction, the solution was made alkaline by adding approximately 5 mL ammonium hydroxide (28.0–30.0%) in an ice bath. The round flask was left in an ice bath for 20 min, and a black precipitate formed when the solution became basic (pH = 8). The precipitate was slowly dissolved in 50 mL of 50:50 Ethanol: H_2_O mixture. The dissolved solution was transferred to a 600 mL beaker on a heat plate with a stir bar and slowly heated to boiling. After 12 min, the heat and stir were turned off, and the product was allowed to cool down to room temperature (r.t). The product was left at room temperature (r.t) to recrystallize over three days. No visible color change was observed. The black product was collected and stored.

### 2.3. Synthesis of Metal Chelator A2

To synthesize A2, Boc-protected iminodiacetonitrile was synthesized by the reaction of iminodiacetonitrile and three equivalents of di-tert-butyl-dicarbonate in a 1:1 dichloromethane, acetonitrile solution. Then, 1.5 equivalents of diisopropylethylamine were added to the reaction mixture, and the reaction was stirred at room temperature for 12 h. The product was purified using a silica gel column with 7:3 hexane/ethyl acetate solvent. For the next step of the reaction, 5 mmol of the synthesized Boc-iminodiacetonitrile and 10 mmol of 2-aminophenol were dissolved in 10 mL of ethylene glycol. The reaction mixture was refluxed for 8 h at 100 °C, cooled to room temperature, and allowed to crystallize. The solid crystal was filtered and dried. Finally, Boc deprotection was done with 1:1 trifluoracetic acid and dichloromethane. The solvent was removed to get the product A2.

### 2.4. Synthesis of Metal Chelator A3

Potassium carbonate (250 mg,1.8 mmol) was suspended in acetonitrile, and A2 was added to it. One equivalent of propargyl bromide (1.8 mmol) was added and a catalytic amount of potassium iodide (0.1 equivalents) was added to the reaction mixture. The reaction was refluxed at 90 °C overnight. The reaction was filtered to remove excess potassium carbonate, and acetonitrile was removed under vacuum. Water was added to the mixture, then the product was extracted in dichloromethane twice. The combined organic phases were washed with brine and dried over anhydrous sodium sulfate to yield A3 (85% yield). The alternate route for A3 synthesis was obtained by modifying a literature method [34]. Commercially available 2-(chloromethyl)-1,3-benzthiazole was reacted with propargyl amine in the presence of a catalytic amount of potassium iodide and 3 equivalents of potassium carbonate in acetonitrile under reflux condition for 12 h. The product intermediate was isolated using solvent extraction to yield A3.

### 2.5. Synthesis of Aβ Inhibitor Peptide LPFFD by Alstra Microwave Peptide Synthesizer

A total of 250 mg of Rink amide resin (loading factor 0.51 mmol/g) was used for the peptide synthesis. The synthesis was performed on the Biotage Alstra Microwave peptide synthesizer, using a customized program. Rink Amide resin was first swelled in NMP at 70 °C for 20 min with an oscillating mixer, then the Fmoc protecting group from the resin was removed by treatment with 20% piperidine in DMF at room temperature for 13 min with an oscillating mixer on an on/off interval of 10 s/15 s. Amino acids were coupled from the C-terminal to the N-terminal. Couplings of Leu, Pro, and Asp were done with the corresponding Fmoc-protected amino acid, Oxyma, and DIC solutions in NMP at 75 °C for 5 min with the oscillating mixer on. For the coupling of two consecutive identical amino acids in the LPFFD sequence, the first Phe was coupled at 75 °C for 5 min, and the second Phe was coupled at 50 °C for 15 min, as per the Alstra synthesizer manual conditions for optimal coupling of two consecutive identical amino acids.

### 2.6. Synthesis of Bifunctional Molecules L1 and L2

Conjugation of LPFFD peptide to bromoacetic acid: LPFFD peptide was swelled in DCM for 90 min before the reaction. In a centrifuge tube, 5 equivalents of bromoacetic acid and 5 equivalents of HOBt were dissolved in 3 mL of 1:9 DCM: DMF mixture. The dissolved solution was added to 100 mg of swelled LPFFD peptide on the resin in solid-phase peptide synthesis (SPPS) tubes (Restek, Bellefonters, PA, USA). Five equivalents of DIC were added to the SPPS tubes to complete the reaction. HOBt and DIC activated the carboxylic group on the bromoacetic acid, while DIC made the reaction faster. The SPPS tubes were placed on the rotor for continuously shaking at r.t for 15 min before the solution was drained. The reaction was repeated for another round by adding bromoacetic acid, HOBt, and DIC to the resin and shaking for 15 min. Next, the resin was washed four times with DCM: DMF (1:9), then with DCM, and dried.

Conjugation of A1 and A2 to brominated LPFFD peptide: 100 mg of each brominated inhibitor peptide from the last step was transferred to a dried 25 mL round bottom flask with a stir bar. In a centrifuge tube, three equivalents of the corresponding metal chelator for each reaction were dissolved in anhydrous DMF. The brominated LPFFD peptide dissolved in anhydrous DMF was added to the round flask. Next, two equivalents of anhydrous 2,6-lutidine were added to the round flask. The round flask was capped with a rubber cap, placed in an oil bath at 45 °C, and stirred for 48 h. Following the reaction, the product was transferred, washed with DMF and DCM, and dried.

### 2.7. Synthesis of Bifunctional Molecule L3

The sidechain-protected LPFFD peptide on rink amide resin was coupled with azido acetic acid. Then, three equivalents of the chelator–alkyne product were added to the azido-containing peptide in the presence of three equivalents of copper(I) iodide and ten equivalents of ascorbic acid in 20% piperidine in DMF for 12 h. Then, the product was transferred into an SPPS tube, and excess copper ion was removed by repeated washes with a chelating solution of 5% sodium diethyl dithiocarbonate and 5% DIEA in DMF [35]. Finally, the resin was washed with DMF and DCM and dried.

### 2.8. TFA Cleavage and Lyophilization

Peptides and bifunctional molecules are cleaved from the resin before characterization by HPLC, LC-MS, and for use in assays. For characterization, only 30–40 mg of dried resin containing the peptide or bifunctional compound was cleaved with a TFA cocktail and characterized. A TFA cleavage solution included 95% TFA, 2.5% TES, and 2.5% H_2_O was prepared for each sample [36]. In a 34 mL glass vial with a stir bar, the resin was added with 1–2 mL of TFA cleavage solution and stirred for 2 h. Afterward, the cleavage solution was pipetted through glass wool to remove the resin, and the filtrate precipitated into cold diethyl ether. The precipitate formed a pellet on centrifugation, and the supernatant ether was discarded. The pellet was resuspended in 2–3 mL of H_2_O. A minimal amount of ACN was added if the pellet did not dissolve completely in water. The dissolved pellet was frozen using liquid nitrogen and lyophilized overnight using a lyophilizer (Labconco, Kansas City, MO, USA).

### 2.9. Purification of Bifunctional Molecule by Reverse-Phase High-Performance Liquid Chromatography

After lyophilization, peptides were subjected to analysis in reverse-phase high-performance liquid chromatography (RP-HPLC), Agilent 1260 using a gradient of HPLC grade H_2_O and ACN, both containing 0.1% TFA. The collected fractions were lyophilized and used for LCMS analysis.

### 2.10. Fluorescence Assays

All metal ions were selected based on their relevance in the biological systems. L1, L2, and L3 solutions were prepared in 10 mM HEPES buffer pH 7.34. The absorbance and fluorescence of the ligand samples were measured with 200 μL of sample added to a high-performance quartz cuvette (200 μL capacity and spectral range 200–1500 nm). For the ligand titration assays, the metal solution was added to the ligand solution in a 1.65 mL mini-centrifuge tube, vortexed to mix the solution, and then transferred to the quartz cuvette. A total of 2 μL of 100× concentrated metal ions solutions were added to the 200 μL of each ligand solution and was added with so that the final peptide: metal was at a 1:1 ratio. For example, for L3, 2 μL of 200μM metal ions were added to a 200 μL solution of 2 μM L3 solution, and so that the final ratio metal-ion: ligand ratio was 1:1. Absorbance and fluorescence measurements were taken using the cuvette module of the SpectraMax M5 Multi-mode microplate reader. L1, L2, and L3 fluorescence intensities were recorded with excitation and emission at 275 nm and 290–600 nm, respectively. The fluorescence was measured immediately after adding and mixing the ions to the ligands L1, L2, and L3 and did not change with time. In the ThT fluorescence assays, the fluorescence intensities were recorded with excitation and emission at 450 nm and 485 nm [26], respectively, and fluorescence was recorded immediately after the addition and mixing of ThT.

### 2.11. Microscopy

Samples prepared for fluorescence assays were used for imaging to visualize how the bifunctional ligand L3 affected the aggregation of Ab42. Following the fluorescence assays, in which the Cu^2+^ was incubated with Ab42 for 48 h at 37 °C and then incubated with ThT for 48 h, they were imaged using ZEISS Microscopy Apotome 3 with 40× magnification. The microscope images were analyzed using ZEN 3.4 (ZEN pro) software. The microscopy images were processed using the same setting on the camera raw filter in Adobe Photoshop, dehaze 90%, clarity 80%, and contrast 30%, settings similar to those suggested to get better microscopy images [37].

## 3. Results and Discussion

### 3.1. Development of Tri-Dentate and Tetra-Dentate Frameworks for Copper Detection

**Figure 1:**
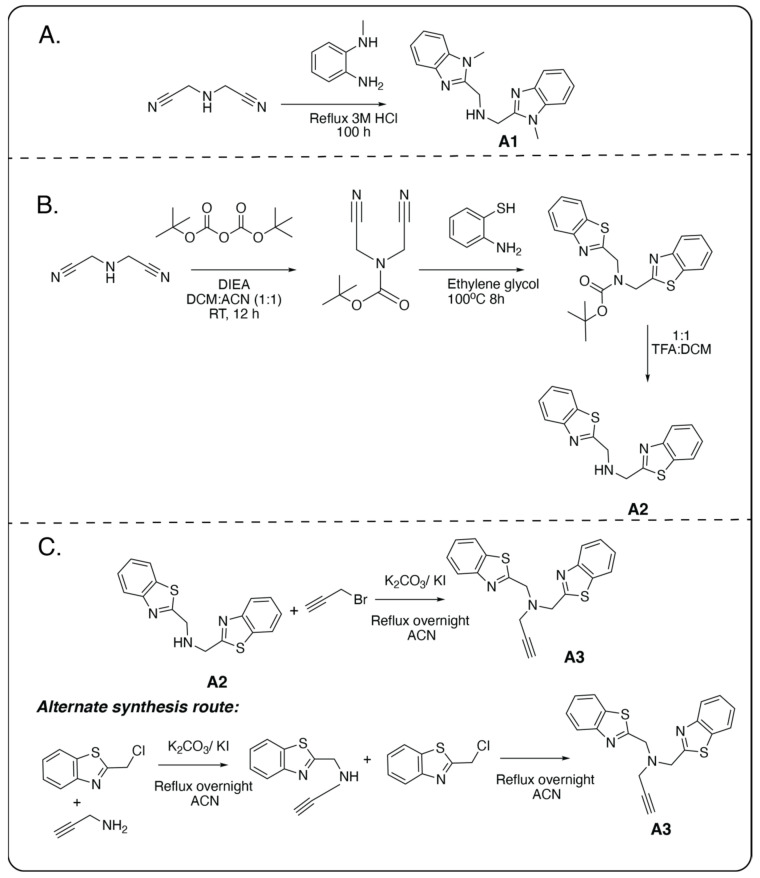
Synthesis scheme for tridentate and tetradentate Cu^2+^-chelator cores. (**A**) Synthesis scheme for core A1. (**B**) Synthesis scheme for core A2. (**C**) Synthesis schemes for core A3, an intermediate of L3.

Three cores were developed for Cu^2+^ chelation (Figure 1). A1 (SI Figures S1 and S2) and A2 (SI Figures S3–S5) incorporated benzimidazole or benzothiazole. A1 was synthesized following a literature protocol [33] (approximate yield of 70%) (Figure 1A). A2 was synthesized by modification of the method used to synthesize A1. A3 were synthesized by modification of a literature method [34] (approximate yield 82% and 80%, respectively) (Figure 1B,C). A3, in addition to the benzothiazole, contained an alkyne that later reacted to form a triazole, which allowed a change in the coordination of Cu^2+^ and increased the framework selectivity towards Cu^2+^. The schemes for synthesis A1, A2, and A3 synthesis are shown in Figure 1. In the first route for A3 synthesis, A2 needed to be synthesized first. Briefly, Boc-protected Iminodiacetonitrile (Figure S3) was synthesized and heated with 2-Aminothiophenol to form its benzothiazole derivative A2 (Figure 1B). To form A3, A2 was reacted with propargyl bromide (Figure 1C). In the alternative scheme to synthesize A3 (Figure 1C), synthesis of A2 was not required, and commercially available materials were used to synthesize A3 (Figure 1C, alternative route). While the first route to synthesize A3 gave a high yield (approximately 80% yield), it required the syntheses of A2 and A3, which involved more steps. The alternate route to synthesize A3, on the other hand, did not require as many steps and could be done using commercially available starting materials, but it did not have as high a yield (approximately 70% yield) as the first route. Characterizations are provided in the Supplementary Information.

### 3.2. Development of Bifunctional Peptide-Framework Hybrid Molecules

**Figure 2:**
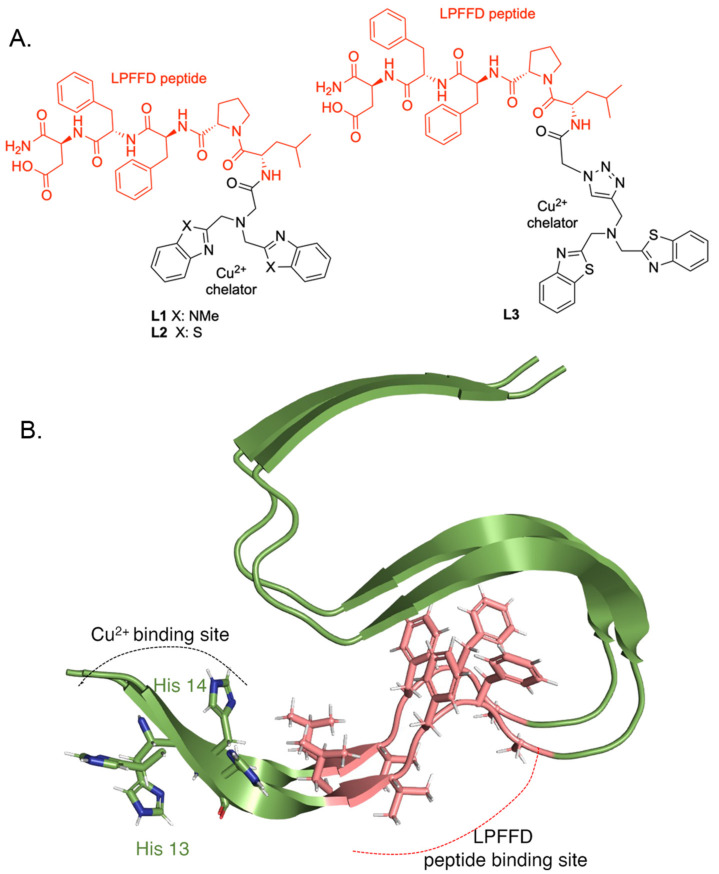
Structure of peptide-metal framework hybrid compounds L1, L2, and L3 and the binding sites of the peptide and Cu^2+^ on Ab42 peptide. (**A**) Bifunctional ligands L1, L2, and L3 consist of two parts—the peptide LPFFD (in red), a known inhibitor of amyloid beta aggregation, and the developed Cu^2+^ chelator cores A1, A2 and A3, respectively (in black). (**B**) The known chelating sites for Cu^2+^, His 13, and His 14 side chains are shown, and their proximity to the central hydrophobic motif (depicted in pink), where LPFFD binds, is shown. PDB ID 2MXU [38] was created using The PyMOL Molecular Graphics System, Version 2.0 Schrödinger, LLC.

Hybrid molecules L1 (SI Figure S6) (yield approximately 30%), L2 (SI Figure S7) (yield approximately 50%), and L3 (SI Figure S8) (yield approximately 75%) were made by conjugating the LPFFD peptide to A1, A2, and A3, respectively, using the SN2 reaction (SI Figures S9–S12) or the CuAAC click reaction (SI Figures S13 and S14). By conjugating a metal ion chelator molecule with an Ab42 inhibitor peptide, we aimed to synthesize bifunctional molecules with metal-chelating and anti-aggregation functions. The Cu^2+^-binding and peptide-binding sites are within 3 amino acids in the Ab42 fibril structure [38] (Figure 2B), which should be compatible with the length of the amide or triazole linkages.

### 3.3. Selectivity of Compounds L1, L2 and L3 towards Copper and Other Physiologically Relevant Metal Ions

For measuring each compound’s fluorescence, a solution of the compound was excited at 275 nm, and the fluorescence emitted was measured from 290 nm to 500 nm. The absorbance spectra of L1, L2, and L3 in 10 mM HEPES buffer pH 7.34 are shown in SI Figure S15. The fluorescence was measured immediately after adding and mixing the ions to the ligands L1, L2, and L3 and did not change with time. SI Figure S16 shows that the fluorescence of the ligand L3 monitored at 290–600 nm, when excited at 275 nm, remained unchanged after 24 h.

To determine the effect of each ion on L1, 2 μL of 2 mM of each metal ion solution was added to 200 μL of 20 μM L1 solution so that the final concentration of each metal ion is 20 μM, to get to a 1:1 equivalent between the ion and L1. It was expected that on the formation of a complex with a metal ion, there would be a change in fluorescence of the L1, L2, and L3 due to metal-induced fluorescence quenching. Quenching of fluorescence by metals can occur following two processes: dynamic quenching, in which there is a decrease in fluorescence due to the physical collisions among molecules, and static quenching, in which fluorophores form nonfluorescent complexes with quenchers [39]. Since the L1, L2, and L3 ligand frameworks were designed to form complexes with Cu^2+^, the decrease in fluorescence would probably be due to static quenching [40] on the complexation of each compound to Cu^2+^. It has previously been reported that Cu^2+^, when coordinating with a benzimidazole derivative (such as L1), causes a decrease in fluorescence [41].

A significant lowering of fluorescence of 20 μM solution of the ligand L1, from 1600 units to 400 units in HEPES buffer pH 7.34, was observed on adding 20 μM Cu^2+^. 20 μM L1 was considered a suitable concentration for the fluorescence assay as it showed a distinct change in fluorescence. Along with the decrease in fluorescence on Cu^2+^ addition, there was a significant lowering of the fluorescence on adding one equivalent of 20 μM Co^2+^ or 20 μM Ni^2+^. Therefore, while L1 preferred binding to Cu^2+^, it was not selective enough because of its interactions with Co^2+^ and Ni^2+^ (SI Figures S17 and S18). Since the 20 μM L1 did not show the desired selectivity for Cu^2+^, no further explorations of the L1 fluorescence at other concentrations were done, and the ligand L2 was explored instead.

On adding 2 μL of 2 mM solutions of various ions to 20 μM L2 solution (to reach a final 1:1 ratio), a significant decrease in fluorescence from 400 units to 300 units was observed for 20 μM Cu^2+^ (SI Figure S19). However, the fluorescence of the 20 μM solution of L2 was surprisingly low (400 A.U.), 4× less than that of the L1 solution (1600 A.U.), and aggregation was also observed at this concentration. The low solubility was not unexpected as benzothiazole has lower solubility than benzimidazole in an aqueous buffer [42]. Since L2, with the benzothiazole core, should have had more fluorescence than L1, containing the benzimidazole core, we hypothesized that self-quenching might be happening, leading to the low fluorescence. It is known that at high concentrations of fluorophore solutions, the fluorescence emission intensity decreases because of self-quenching [43,44]. Self-quenching can occur via various mechanisms, such as collisions between excited fluorophores, the formation of non-fluorescent dimers, and energy transfer to the nonfluorescent dimers [43,45,46].

Therefore, to verify if L2 had self-quenching and if using lower concentrations of L2 would help us avoid the problem, the L2 solution was diluted to 8 μM, and for this less concentrated solution, we observed an increased fluorescence (800 A.U.) (SI Figure S20). On titration with various metal ions with the 8 μM L2 solution in 10 mM HEPES buffer pH 7.34, the distinct lowering of fluorescence only for Cu^2+^ was no longer observed. The fluorescence intensity decreased to different extents for the various ions, with Cu^2+^ addition resulting in the most significant change (SI Figure S20). The L2 solution was further diluted to 0.2 μM, and its fluorescence was measured. The 0.2 μM L2 solution gave a fluorescence of 600 A.U., and on titration with different metal ions, the 0.2 μM L2 solution showed a trend similar to the 8 μM L2 solution, i.e., a lowering of fluorescence to different extents (SI Figure S21). Therefore, while L2 contained the more fluorescent core of benzothiazole, it was unsuitable as a selective Cu^2+^ sensor as it bound Cu^2+^ and other metal ions.

**Figure 3:**
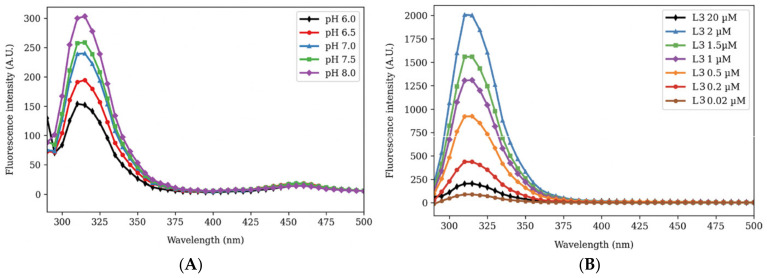
Fluorescence of L3 at different pH and different concentrations. (**A**) Fluorescence intensities of 20 μM L3 differed at different pH values from 6.0 to 8.0, and higher fluorescence intensities were observed at pH values of 7–8. The highest fluorescence was seen at pH 8.0, but high fluorescence was also seen at pH 7.5, close to the pH of 7.34 used in experiments. (**B**) Fluorescence intensity of L3 changed at different concentrations from 20 μM to 20 nM at pH 7.34. The highest fluorescence intensity was observed with 2 μM L3 (blue line), while 20 μM L3 was self-quenched and had a low fluorescence intensity of about 220 A.U. (black line).

To increase the selectivity of the ligand for Cu^2+^, we designed a TBTA-like structure [47] for the next ligand L3. L3 should have 4 coordination sites preferred by Cu^2+^ over three-coordination sites like L1 and L2. We observed that the L3 had the highest fluorescence in the pH 7 to 8 range, so a pH of 7.34 was suitable for the fluorescence assay (Figure 3A, bar diagram SI Figure S22). For the 20 μM L3 solution, we observed a fluorescence of 250 units, which decreased to 70 units with the addition of 20 μM Cu^2+^ (SI Figure S23). L3, which contained a benzothiazole core and a triazole core, should have more fluorescence than L1 (benzimidazole core) and fluorescence similar to L2 (benzothiazole core). Therefore, we suspected that L3 was undergoing self-quenching at high concentrations like L2, and we did dilutions to study whether the fluorescence increased with dilution. Like L2, the fluorescence of L3 increased with dilution, and therefore, we monitored the fluorescence of a dilution series of the ligand L3 (Figure 3B, bar diagram SI Figure S24). Figure 3B shows that the fluorescence of L3 can be detected at 200 nM concentration (400 A.U. fluorescence, red line in Figure 3B). Based on this series, it was decided that the titration against ions would be done using a 2 μM L3 solution, as that would allow the detection of fluorescence change over a wide range.

**Figure 4:**
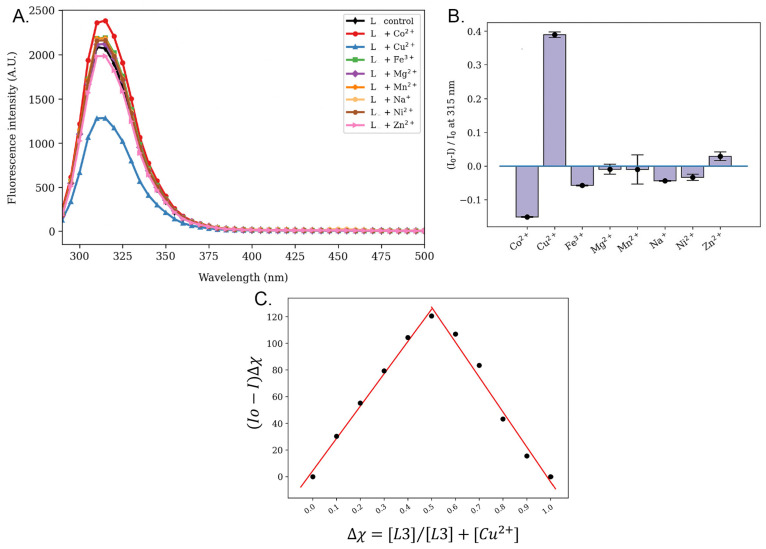
L3 selectivity towards different metal ions and Job’s plot for L3 binding to Cu^2+^. (**A**) The fluorescence changes of 2 μM L3 with different metal ions were measured from 290 to 500 nm. 2 μM L3 in HEPES buffer pH 7.34 had an intensity at around 2000 A.U. (black line). Adding 2 μM Cu^2+^ decreased the intensity to around 1000 A.U. (blue line). Adding other metal ions did not lead to any such significant change. (**B**) Normalized fluorescence change of 2 μM L3 with different metal ions at the 315 nm peak. The binding of Cu^2+^ led to the most significant change in fluorescence intensity, which was normalized. Changes for all other metal ions were expressed as a fraction of the largest change. The samples were done in triplicate. (**C**) Job’s plot diagram of the formation of Cu^2+^ complex with L3. Δχ was the mole fraction of L3 and (I_o_-I) was the emission intensity change.

The fluorescence of 2 μM L3 in buffer pH 7.34 was ~2000 A.U., and with the addition of 2 μM copper (black line), fluorescence was significantly lowered to ~1250 A.U. (Figure 4A). In contrast, there was no such effect on the addition of 2 μM Fe^3+^, 2 μM Mg^2+^, 20 μM Mn^2+^, 2 μM Na^+^, 2 μM Ni^2+^ or 2 μM Zn^2+^, while the addition of 2 μM Co^2+^ led to a slight increase of fluorescence by ~300 A.U.—this could be due to the oxidation of Co^2+^ to Co^3+^. Therefore, we could confirm that L3 was a selective Cu^2+^ binder and did not react significantly with other ions in the 10 mM HEPES buffer at pH 7.34 (Figure 4B).

To examine the binding ratio between L3 and Cu^2+^, a Job’s plot experiment of L3 with Cu^2+^ was performed in 10 mM HEPES buffer pH 7.34 (Figure 4C). Varying volumes (from 0 μL to 250 μL) of 2 μM Cu^2+^ solution were titrated into varying volumes (from 250 μL to 0 μL) of 2 μM L3 solution, so the ratio of L3 and Cu^2+^ varied, but the total volume of the mixed solution (250 μL) was constant. Job’s plot [48] was drawn by plotting (Io-I)Δχ vs. χ, where (Io-I) was the change in fluorescence intensity in the spectrum during titration and χ was the mole fraction of the host, the ligand L3. When the equivalents of the ligand L3 were equal to the equivalents of Cu^2+^, the change in fluorescence times fraction of L3 reached its peak, indicating that the binding of L3 and Cu^2+^ was optimal at a 1:1 ratio.

L3 was selected over L1 and L2 for studying the amyloid aggregation in the presence of Cu^2+^, as it showed selectivity for Cu^2+^ over other metal ions, and therefore had the potential to inhibit amyloid beta- Cu^2+^ aggregation with better selectivity. Further characterizations of L1 and L2 were not done, and we moved on to studying the effect of L3 over amyloid beta aggregation in the presence of Cu^2+^.

### 3.4. Effect of L3 on Cu^2+^-Ab42 Aggregation

Before investigating the effect of L3 on Cu^2+^-Ab42 aggregation, we studied the effect of Cu^2+^ addition on Ab42 aggregation. The fluorescence of Ab42 peptide in 10 mM HEPES buffer pH 7.34 was monitored by monitoring the tyrosine fluorescence. A total of 20 μM Ab42 peptide in 10 mM HEPES buffer, pH 7.34, was incubated for 48 h at 37 °C following literature protocol [49], and the fluorescence at 310 nm, on excitation at 280 nm, was monitored. The peptide showed fluorescence from 290 nm to 360 nm with a maximum intensity of 800 A.U. Then, Cu^2+^ was added to the Ab42 solution in a 1:1 ratio and incubated for 48 h at 37 °C, following a literature protocol [26]. The fluorescence at 310 nm was monitored at the end of the incubation period. As seen in SI Figure S25, the fluorescence of Ab42 at 310 nm was completely diminished after incubation with Cu^2+^, thereby showing the fluorescence quenching effect. The fluorescence quenching effect is due to the paramagnetic nature of the Cu^2+^ complex [50]. The reduction of Cu^2+^ to Cu^1+^ can induce the formation of reactive oxidative species that cause the formation of tyrosine cross-links by covalent ortho–ortho coupling of two tyrosine residues. A total of 310 nm fluorescence monitoring did not help study the effect of L3 on Cu^2+^–Ab42 because L3 had fluorescence at 310 nm, and even if the addition of L3 caused less quenching of Cu^2+–^Ab42, it would not have been easy to distinguish the effect from the fluorescence of L3 itself. The traditional assay of monitoring tyrosine fluorescence that had previously been successfully used to determine the binding of ligand to Ab in the presence of Cu^2+^ [26] was, therefore, unsuitable for our system.

**Figure 5:**
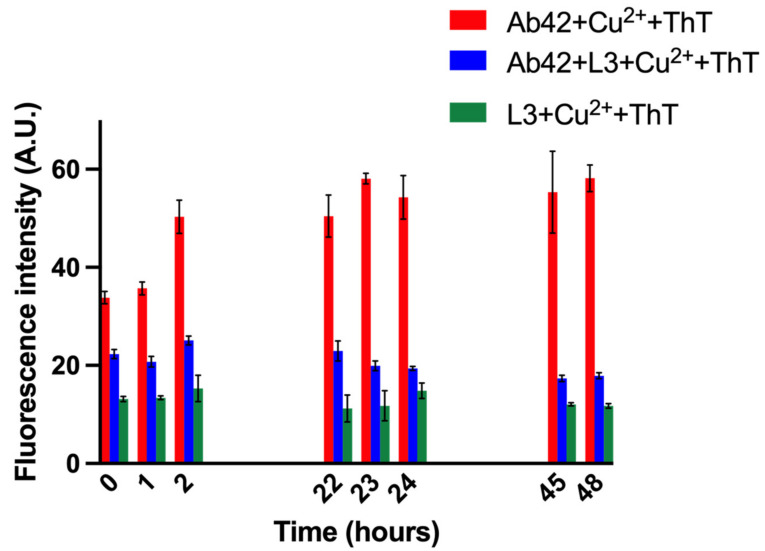
Monitoring aggregation of amyloid beta-copper solutions by ThT fluorescence in the presence of L3. ThT fluorescence for the equimolar 20 μM Ab42-Cu^2+^ increased initially and then became constant (red bars), whereas, in the presence of 1 equivalent of L3, the lower ThT fluorescence intensity remained constant (blue bars). L3-Cu^2+^, in the absence of amyloid peptide, interacted with ThT to produce a low constant background (green bars). Samples were done in triplicate.

Therefore, we explored the widely used assay for amyloid beta aggregation, by monitoring Thioflavin T (ThT) fluorescence [26]. ThT binding to Ab42 fibrils causes high fluorescence. However, other non-fibrillar aggregates of Ab42, such as soluble aggregates have also been monitored successfully with ThT fluorescence [51,52]. When ThT is excited at 450 nm, it emits fluorescence at 470 nm–700 nm, and ThT fluorescence is monitored at 485 nm. When Ab42 aggregates, ThT fluorescence increases. Therefore, the more the aggregation of the Ab42-Cu^2+^ sample, the higher the ThT fluorescence. Adding an inhibitor of Ab42 aggregation should cause the ThT fluorescence to decrease. In our experiment, Cu^2+^ was added to the 10 mM HEPES buffer solution of the commercially available Ab42 to represent the physiologically relevant concentration (10 μM), and this solution was used to study the effect of L3. For studying the effect of L3 on Cu^2+^-Ab42 aggregation, 10 μM L3 was added to 10 μM Ab42 and 10 μM Cu^2+^ at pH 7.34 and incubated with light shaking for 22 h at 37 °C. A sample of 10 μM Ab42 and 10 μM Cu^2+^ at pH 7.34, without any L3 addition, was treated identically. ThT was added to each sample, and fluorescence was measured (0 h timepoint). The control consisted of ThT in pH 7.34 buffer, which showed minimal fluorescence (around 1 A.U.) (SI Figure S26, blue bars). For the Ab42-Cu^2+^ containing samples, with and without L3, fluorescence was higher immediately after ThT addition, confirming that the fluorescence detected was due to the ThT binding to the Ab42 aggregates in the presence of Cu^2+^. The fluorescence intensity for ThT continued to increase over 48 h for the equimolar 10 μM Ab42–Cu^2+^ mixture (1:1 ratio) (SI Figure S26, black bars), whereas for the L3-containing Ab42 + Cu^2+^ sample (1:1:1 ratio), the fluorescence increased to around 12 A.U. around 22 h, and then remained constant (SI Figure S26, red bars), which indicated that L3 inhibited the formation of Cu^2+^-Ab42 aggregates. This experiment was repeated in triplicate with 20 μM Ab42 mixed with 20 μM Cu^2+^ for 22 h at 37 °C in HEPES buffer pH 7.34, with or without L3, and then treated with ThT (0 time point). ThT fluorescence monitored over time showed that the fluorescence of the Ab42-Cu^2+^ solution was getting high to around 55 A.U at 2 h and more or less remained constant (Figure 5, red bars) after that time. On the other hand, when L3 was present in the Ab42-Cu^2+^ solution mixed at a 1:1:1 ratio, ThT fluorescence more or less remained steady at around 20 A.U. over time (Figure 5, blue bars). When 20 μM L3 was incubated with 20 μM Cu^2+^ solution in the absence of Ab42 for 22 h at 37 °C, and then ThT was added, an initial fluorescence around 13 A.U. was observed, which remained constant over time (Figure 5, green bars). Therefore, there is a constant background interaction of L3-Cu^2+^ 1:1 mixture with ThT, but L3 decreased Ab42-Cu^2+^ aggregation, as evident from Figure 5 blue bars.

It is known from the literature that ThT with Cu^2+^ does not produce fluorescence [53]. To ensure that the decrease in fluorescence in the presence of L3 did not arise from other interactions, another experiment was run in which 20 μM Ab42, 20 μM Ab42 + L3 (1:1 ratio), and 20 μM L3 were incubated with light shaking for 22 h at 37 °C in 10 mM HEPES buffer pH 7.34, and then treated with ThT and its fluorescence was monitored (SI Figure S27). It was observed that 20 μM Ab42, in the absence of Cu^2+^, underwent aggregation, as ThT fluorescence increased initially to around 43 A.U. around 2 h and then remained constant (SI Figure S27, red bars). However, the ThT fluorescence of 20 μM Ab42 in the presence of 20 μM L3 was reduced and remained constant (SI Figure S27, blue bars). Therefore, L3 bound to Ab42 (even in the absence of Cu^2+^) and inhibited aggregation of Ab42. L3 also produced a low fluorescent background with ThT that remained constant (SI Figure S27, black bars). Combining the observations from Figure 5 and this experiment, we could conclude that L3 could inhibit aggregation of both Ab42 and Ab42 with Cu^2+^.

To check the dose dependence of L3, we treated Ab42-Cu^2+^ with different concentrations of L3 and incubated them for 22 h at 37 °C in 10 mM HEPES buffer pH 7.34, added ThT, and monitored fluorescence over time. Generally, Ab42 must be used at higher concentrations (10 μM or higher) to show significant fluorescence in the ThT assay. Therefore, 20 μM Ab42 was used in the assay. A total of 10 μM L3 and 20 μM L3 were mixed with 20 μM Ab42 and 20 μM Cu^2+^ so that the ligand to receptor (L3 to Ab42-Cu^2+^) ratio was 1:2 and 1:1, respectively (SI Figure S28). In the absence of L3, the Ab42-Cu^2+^ formed aggregates initially and then reached a plateau (SI Figure S28, red bars). The presence of 10 μM L3 decreased the fluorescence significantly (L3:Ab42–Cu^2+^ ratio of 1:2), further confirming the L3 inhibits aggregation of Ab42–Cu^2+^ (SI Figure S28, blue bars). It was observed that a higher ratio of L3 (1:1) caused more lowering of fluorescence, as expected (SI Figure S28). A high fluorescence was observed when L3 was added at a higher ratio than the Ab42-Cu^2+^ solution (3:1 or 4:2), and ThT fluorescence was measured (not shown). This was probably due to the aggregation of L3 at concentrations higher than 20 μM. Future investigation will determine what phenomenon occurs when L3 is present at a greater percentage than Ab42 + Cu^2+^.

**Figure 6:**
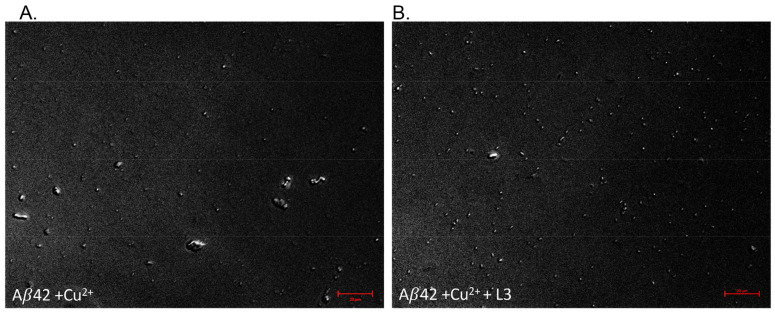
The brightfield image of amyloid beta- Cu^2+^ solution in the presence and absence of L3 48 h after ThT addition. The amyloid beta- Cu^2+^ solution (**A**) formed large aggregates with an average area of 17.3 μm^2^ and some smaller aggregates, while the L3-containing solution (**B**) contained much smaller aggregates of around 2.17 um^2^.

To ensure that the decrease in ThT fluorescence was not erroneously interpreted and corresponded to a decrease in non-amyloid aggregate formation, DIC microscopy was used to visualize the aggregates with and without the ligand L3 (Figure 6). Without the L3 ligand, when the Ab42–Cu^2+^ sample was incubated for 48 h and then treated with ThT for 48 h, a few large aggregates with an average area of 17.3 μm^2^ were observed, along with smaller aggregates (Figure 6A). It has been reported that Cu^2+^ mediates non-amyloidogenic aggregation of Ab40 at the equimolar ratio and that fibrils are not formed at a 1:1 ratio of Cu^2+^ and Ab40 [52]. Our observations and fluorescent intensity values obtained for Ab42 agreed with this previous report. The size of the large aggregates we observed (17.3 μm^2^ area) was much larger than the one reported (>1 μm) for Ab40 [52], while some others have reported large aggregates similar in size without Cu^2+^ [53]. The sample with L3 added to Ab42–Cu^2+^ during the initial incubation for 48 h, then used for microscopy 48 h after ThT addition, did not show large aggregates but smaller soluble aggregates (Figure 6B). This indicated that the L3 ligand inhibited the continued size increase of Ab42–Cu^2+^ aggregates. The control experiment with Ab42 without Cu^2+^ (SI Figure S29A) showed much smaller aggregates than Ab42–Cu^2+^ [54], further confirming the reported literature that Cu^2+^ promotes the formation of larger non-amyloidogenic aggregates of Ab42 [52,53]. Ab42, when incubated with L3 in a 1:1 ratio in the absence of Cu^2+^, showed small aggregates like Ab42 incubated alone (SI Figure S29B). While the ThT fluorescence assay on Ab42 aggregation in the absence of Cu^2+^ (Figure S27) clearly showed the difference between Ab42 and Ab42-L3 and indicated that L3 successfully inhibited Ab42 aggregate formation even in the absence of copper, the corresponding microscope images of Ab42 and Ab42-L3 in the absence of Cu^2+^ (SI Figure S29) did not show any such difference.

## 4. Conclusions

We have successfully designed and synthesized three tridentate and tetradentate Cu^2+^ chelators and incorporated these into hybrid metal–chelator peptide complexes. These complexes were water-soluble, which allowed us to use these as Cu^2+^ sensors under aqueous conditions with a physiologically relevant pH of 7.34. We demonstrated that the newly developed complex L3 was selective for Cu^2+^ binding and bound Cu^2+^ at a 1:1 ratio. We studied the effect of this new complex on non-amyloidogenic soluble aggregates of a 1:1 mixture of Ab42 and Cu^2+^ using fluorescence assays and microscopy. We demonstrated that L3 inhibited aggregation and prevented the formation of very large aggregates of Ab42–Cu^2+^. It is known that micromolar concentrations of Cu^2+^ (up to 400 μM) are present in senile plaques in AD brains [21] and that Cu^2+^ causes non-amyloidogenic aggregates of Ab42 to form at an equimolar ratio [52]. Given that Ab42 aggregates are toxic [51] and accumulate early before insoluble plaque formation and cause cognitive impairment in Alzheimer’s disease [55], the hybrid L3 molecule can potentially be used to slow down the progress of the disease.

## Data Availability

Data are contained within the article and Supplementary Materials.

## Supplementary Materials

The following supporting information can be downloaded at: https://www.mdpi.com/article/10.3390/bios14050247/s1, Figure S1, ^1^H and ^13^C NMR of A1 metal-chelator, Figure S2, HPLC and Mass spectrometry characterization of A1 metal-chelator, Figure S3, ^1^HNMR of Boc-protected Iminodiacetonitrile, Figure S4, Mass spectroscopy characterization of Boc-protected A2, Figure S5, ^1^H NMR of chelator A2 in CDCl_3_, Figure S6, Characterization of L1 by HPLC and MS, Figure S7, Characterization of L2 by HPLC and MS, Figure S8, Characterization of L3 by HPLC and MS, Figure S9, Synthesis of L1 on resin by amide coupling reaction, Figure S10, Cleavage of bifunctional ligand L1 from resin, Figure S11, Synthesis of L2 on rink amide resin, Figure S12, Cleavage of bifunctional ligand L2 from resin, Figure S13, Synthesis of L3 on rink amide resin, Figure S14, Cleavage of L3 from resin, Figure S15, Absorbance spectra of L1, L2 and L3, Figure S16, L3 solution fluorescence intensity constant over 24 h, Figure S17, Selectivity comparison of 20 μM L1 for various metal ions, Figure S18, Normalized fluorescence of L1 on adding different metal ions at a 1:1 ratio, Figure S19, Selectivity comparison of 20 μM L2 for various metal ions, Figure S20, Selectivity comparison of 8 μM L2 for various metal ions, Figure S21, Selectivity comparison of 0.2 μM L2 for various metal ions, Figure S22, Variation of fluorescence intensity of L3 with pH change, Figure S23, Selectivity comparison of 20 μM L3 for various metal ions, Figure S24, Variation of fluorescence intensity with concentration of L3, Figure S25, Ab42 tyrosine fluorescence at 310 nm in the absence of and presence of Cu^2+^, Figure S26, ThT fluorescence assay of 10 μM Ab42 in the presence of 10 μM Cu^2+^ and 10 μM L3, Figure S27, ThT fluorescence assay of 20 μM Ab42 and 20 μM L3 in absence of Cu^2+^, Figure S28, ThT fluorescence assay of 20 μM Ab42 and 20 μM Cu^2+^ in the presence of 10 μM and 20 μM L3, Figure S29, Bright field image of Ab42 and Ab42 and L3.
