# Supplementary material for: A Copper-Selective Sensor and Its Inhibition of Copper-Amyloid Beta Aggregation
Source: Biosensors (Basel). 2024 May 14;14(5):247. doi: 10.3390/bios14050247 (PMC11117483; doi:10.3390/bios14050247)

SUPPLEMENTARY INFORMATION

A copper-selective sensor and its inhibition of copper-amyloid beta aggregation

Ngoc Kim Nguyen, Bella Poduska, Mia Franks, Manoranjan Bera, Ian McCormack, Guoxing Lin, Alexander P. Petroff, Samir Das, Arundhati Nag*

Carlson School of Chemistry and Biochemistry, Clark University, Worcester, MA

Table of figures:

Figure S-1: ^{1}H and ^{13}C NMR of A1 metal-chelator. 3
Figure S- 2: HPLC and Mass spectrometry characterization of A1 metal-chelator 5
Figure S- 3: ^{1}HNMR of Boc-protected Iminodiacetonitrile 6
Figure S- 4: Mass spectroscopy characterization of Boc-protected A2. 7
Figure S- 5: ^{1}H NMR of chelator A2 in CDCl3 8
Figure S- 6: Characterization of L1 by HPLC and MS 9
Figure S- 7: Characterization of L2 by HPLC and MS. 10
Figure S- 8: Characterization of L3 by HPLC and MS 11
Figure S- 9: Synthesis of L1 on resin by amide coupling reaction 12
Figure S- 10: cleavage of bifunctional ligand L1 from resin 14
Figure S- 11: Synthesis of L2 on rink amide resin 15
Figure S- 12: Cleavage of bifunctional ligand L2 from resin 16
Figure S- 13: Synthesis of L3 on rink amide resin 17
Figure S- 14: Cleavage of L3 from resin 18
Figure S- 15: Absorbance spectra of L1, L2 and L3 19
Figure S- 16: L3 solution fluorescence intensity constant over 24 hours. 20
Figure S- 17: Selectivity comparison of 20 µM L1 for various metal ions. 21
Figure S- 18: Normalized fluorescence of L1 on adding different metal ions at a 1:1 ratio. 22
Figure S- 19: Selectivity comparison of 20 µM L2 for various metal ions. 23
Figure S- 20: Selectivity comparison of 8 µM L2 for various metal ions 24
Figure S- 21: Selectivity comparison of 0.2 µM L2 for various metal ions 25
Figure S- 22: Variation of fluorescence intensity of L3 with pH change. 26
Figure S- 23: Selectivity comparison of 20 µM L3 for various metal ions 27
Figure S- 24: Variation of fluorescence intensity with concentration of L3 28
Figure S- 25: Ab42 tyrosine fluorescence at 310 nm in the absence of and presence of $Cu^{2+.}$ 29
Figure S- 26: ThT fluorescence assay of 10 µM Ab42 in the presence of 10 µM Cu^{2+} and 10 µM L3 30
Figure S- 27: ThT fluorescence assay of 20 µM Ab42 and 20 µM L3 in absence of Cu^{2+} 31
Figure S- 28: ThT fluorescence assay of 20 µM Ab42 and 20 µM Cu^{2+} in the presence of 10 µM and 20 µM L3 32
Figure S- 29: Bright field image of Ab42 and Ab42 and L3 33

Figure S-1: ^{1}H and ^{13}C NMR of A1 metal-chelator.

A. ^{1}H NMR of metal chelator A1 in $CDCl_3$. The reference was generated in DMSO at 300 MHz, Observed – ^{1}H NMR ($CDCl_3$): 3.47 (m, N-Me-H), 3.78 (m, N-CH_2-C), 7.2-7.3 (m, benzimidazolyl-H).

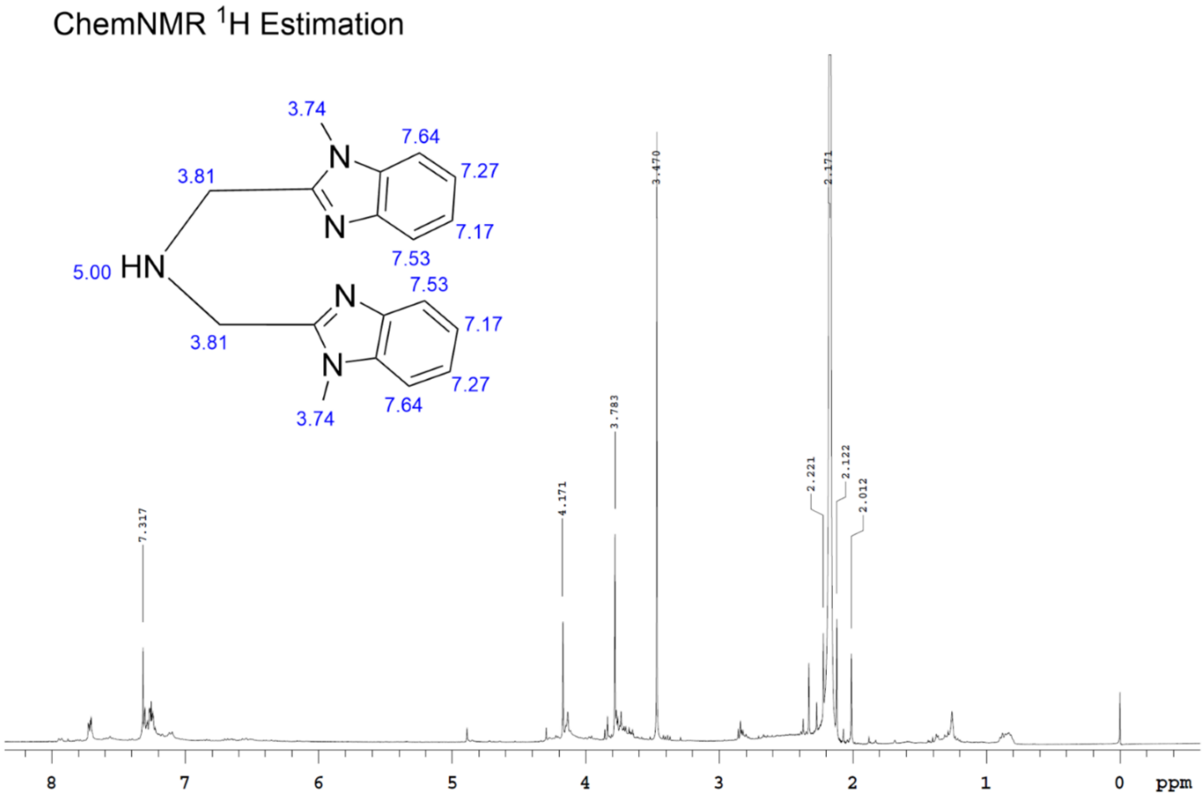

B. ^{13}C NMR of metal chelator A1 in $CDCl_3$. The reference was generated in DMSO at 300 MHz, observed – ^{13}C NMR ($CDCl_3$): 80 ($CDCl_3$), 47.1 (CH_2-C-NH), 148.1 (N-C-N), 31.7 (N-C-H_3), 135.9 (H_3CN-C), 142.2 (N-C-C).

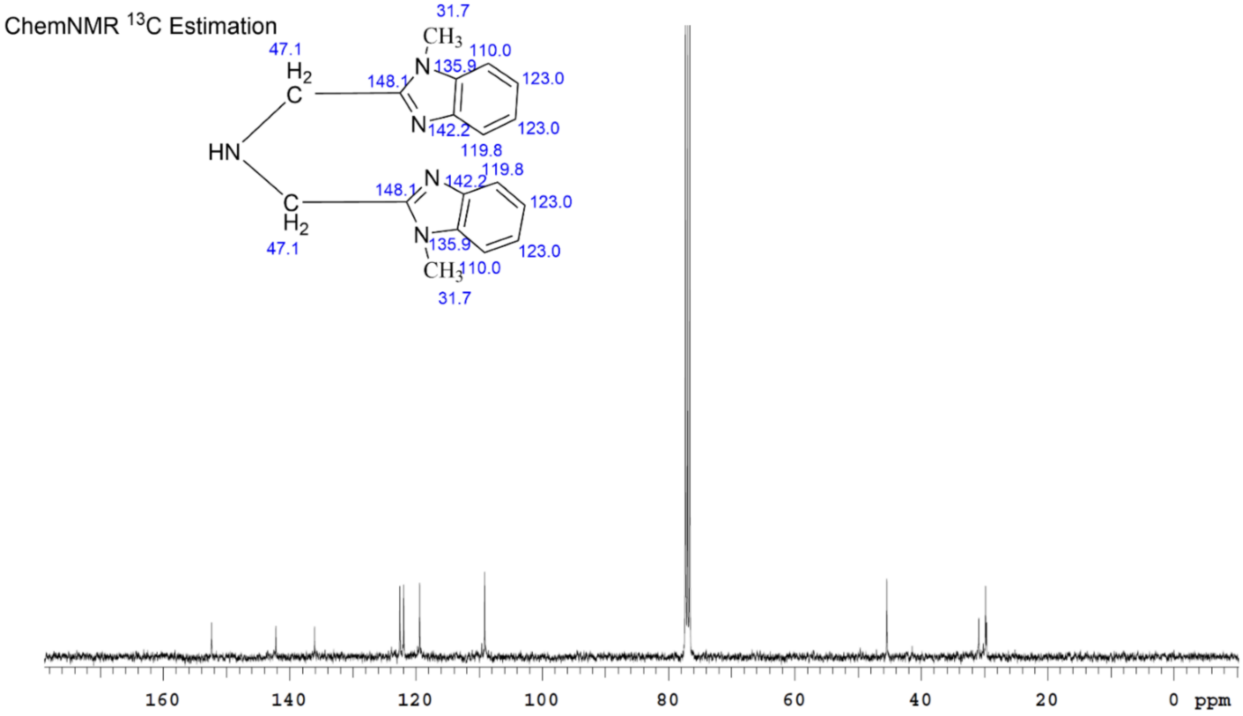

Figure S- 2: HPLC and Mass spectrometry characterization of A1 metal-chelator

A. The HPLC chromatogram of A1 detected at 280 nm with one major peak at 12 minutes (arrow). The. peak has an intensity of 80 mAU. The arrow points to the major peak that was further analyzed in the LCMS. B. MS of the collected peak confirms the correct mass of chelator A1 with one proton (306.16 Da).

A.

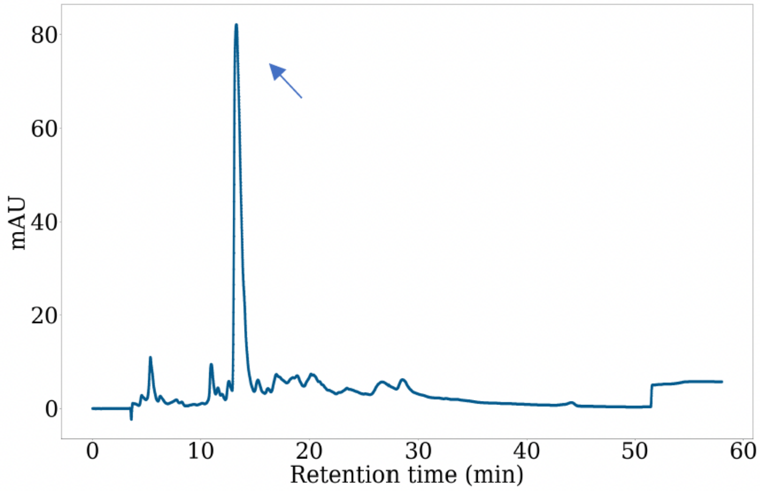

B.

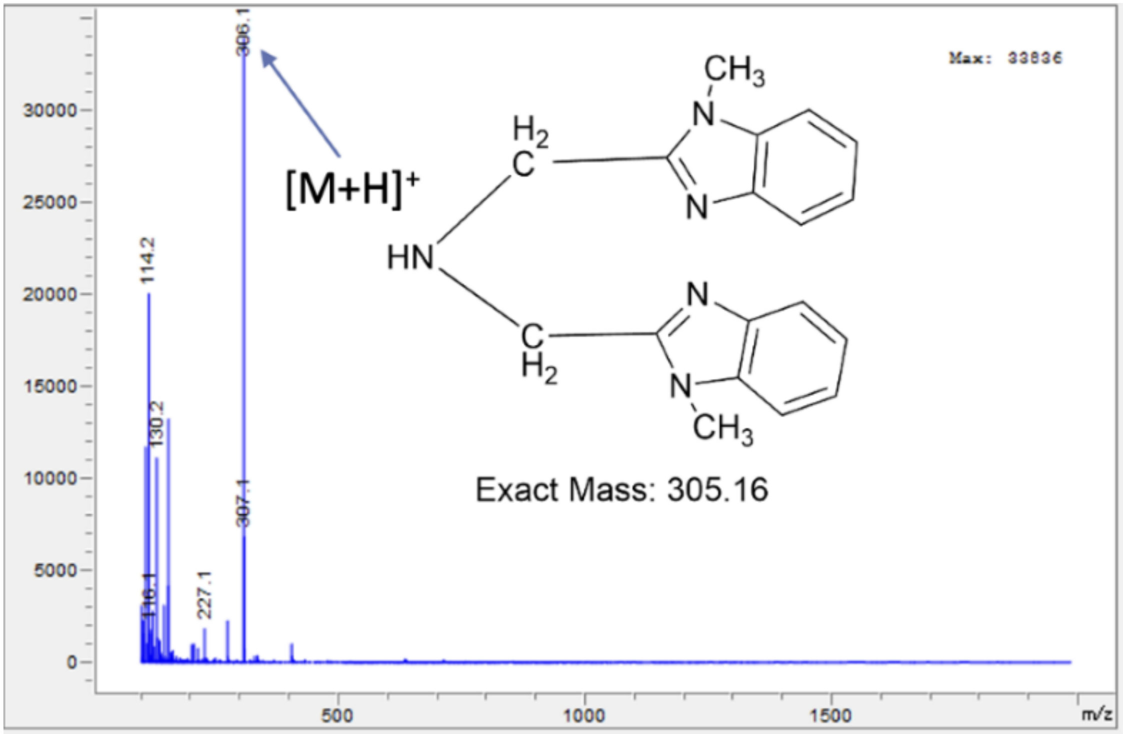

Figure S- 3: ^{1}HNMR of Boc-protected iminodiacetonitrile

^{1}H NMR of Boc-protected iminodiacetonitrile in $CDCl_3$. The reference was generated in DMSO at 300 MHz – ^{1}H NMR ($CDCl_3$): d (ppm) 4.29 (m, 2H, ≡CN), 1.44 (m, 3H).

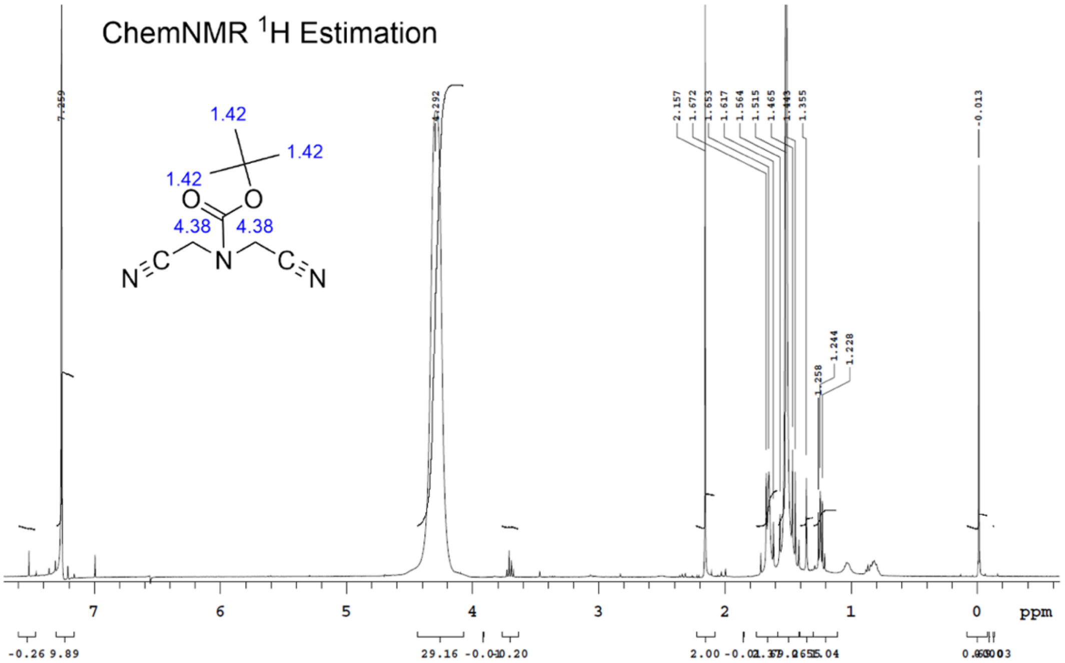

Figure S- 4: Mass spectroscopy characterization of Boc-protected A2.

The correct mass with one proton (412.0 Da) was detected in the MS.

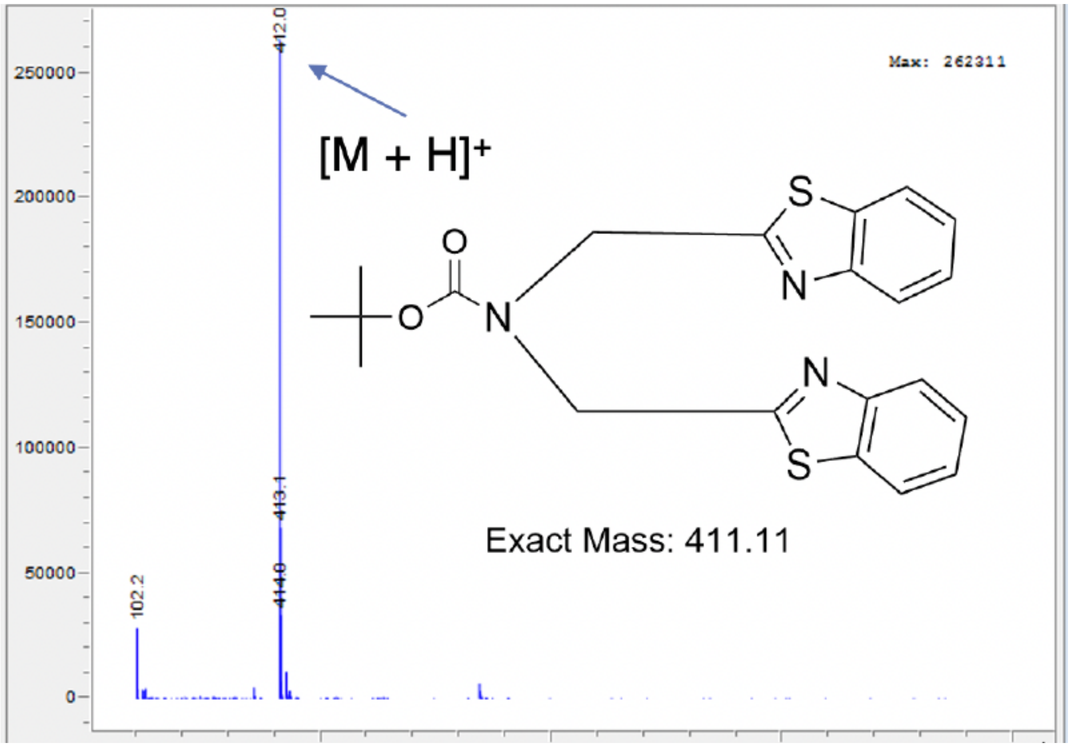

Figure S- 5: ^{1}H NMR of chelator A2 in $CDCl_3$

^{1}H NMR ($CDCl_3$): d (ppm) 4.71 (m, 2H, -N), 7.25-7.98 (m, benzimidazolyl-H).

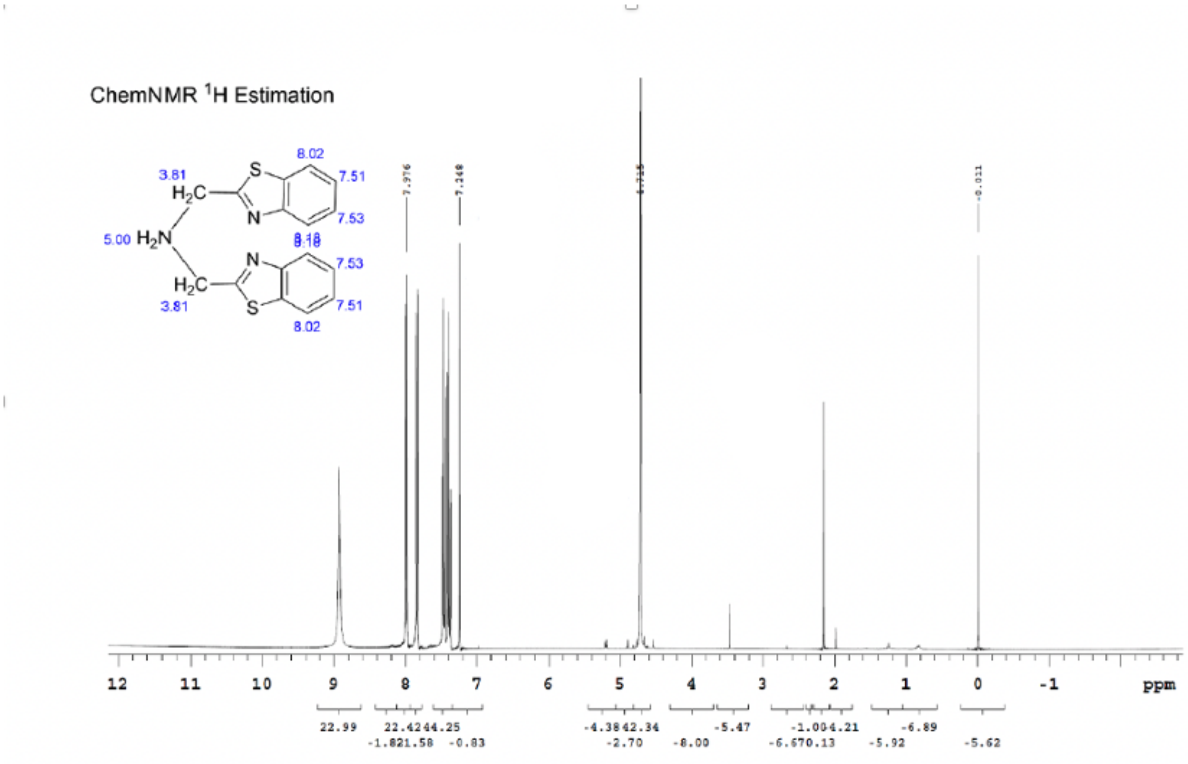

Figure S- 6: Characterization of L1 by HPLC and MS

A. The RP-HPLC of synthesized L1 shows the major peak at 38.7 minutes. B. MS analysis of the 38.7 min. RP-HPLC peak shows correct L1 mass with z = 1(983.5 Da) and z = 2 (491.9 Da).

A.

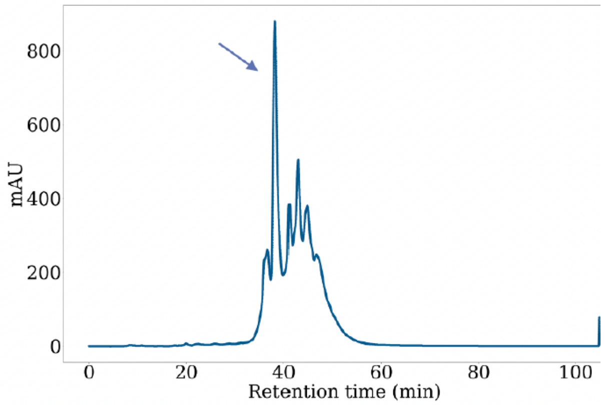

B.

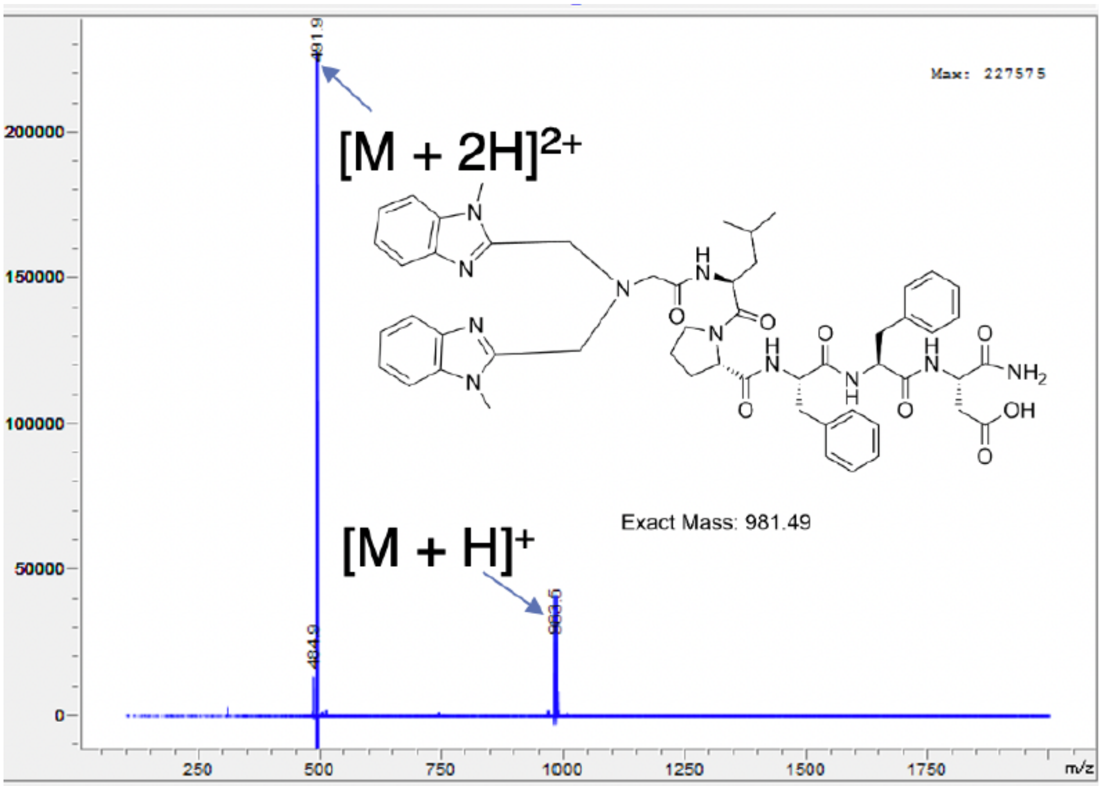

Figure S- 7: Characterization of L2 by HPLC and MS.

A. The major peak of the RP-HPLC was at 43 minutes. B. The correct mass of the L2 was obtained by analyzing the major peak in the MS.

A.

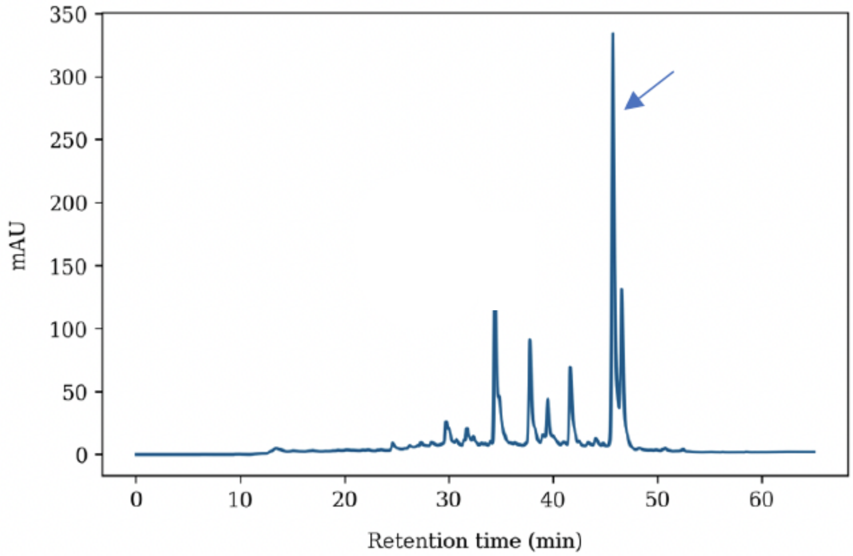

B.

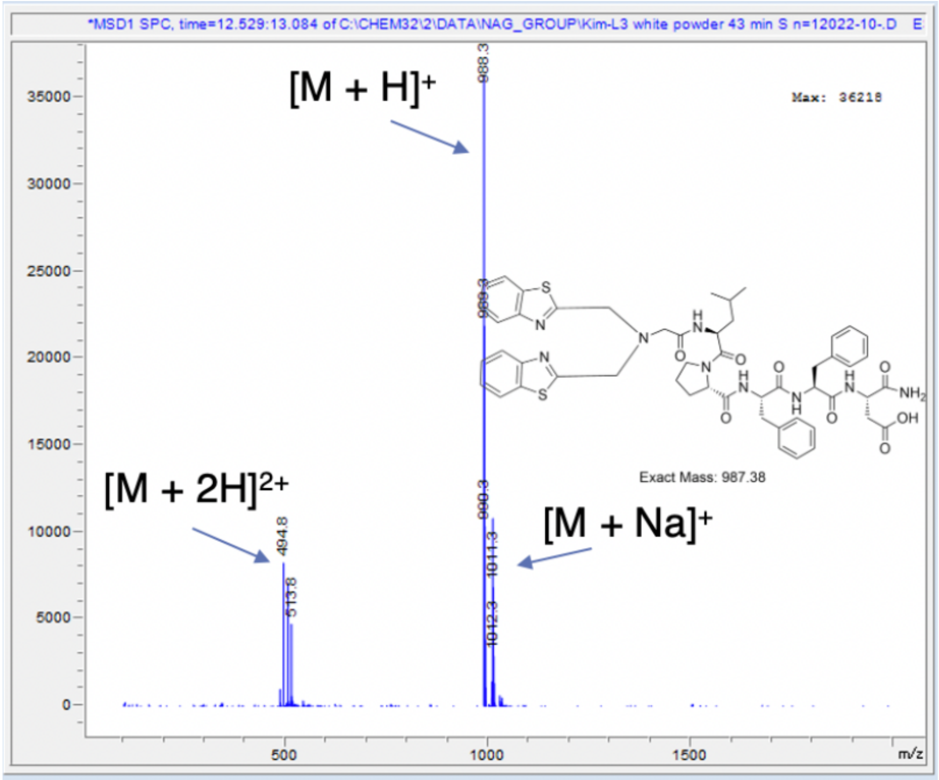

Figure S- 8: Characterization of L3 by HPLC and MS

A. The major product eluted at 31 minutes during the RP-HPLC purification (280 nm). B. The correct mass of the L3 was obtained from the 31-minute HPLC peak as $(M+H^{+})$ and $(M+2H)^{2+}$.

A.

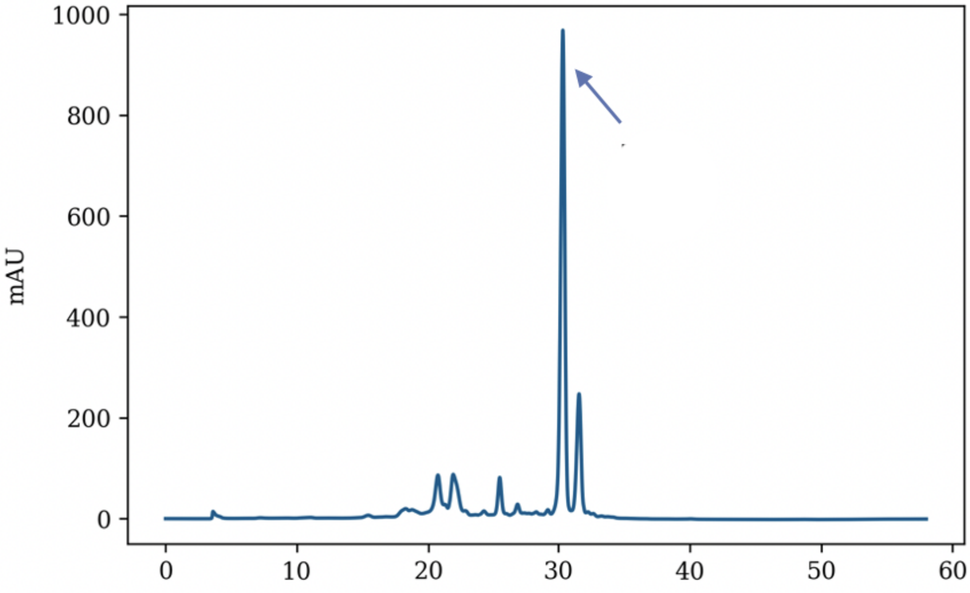

B.

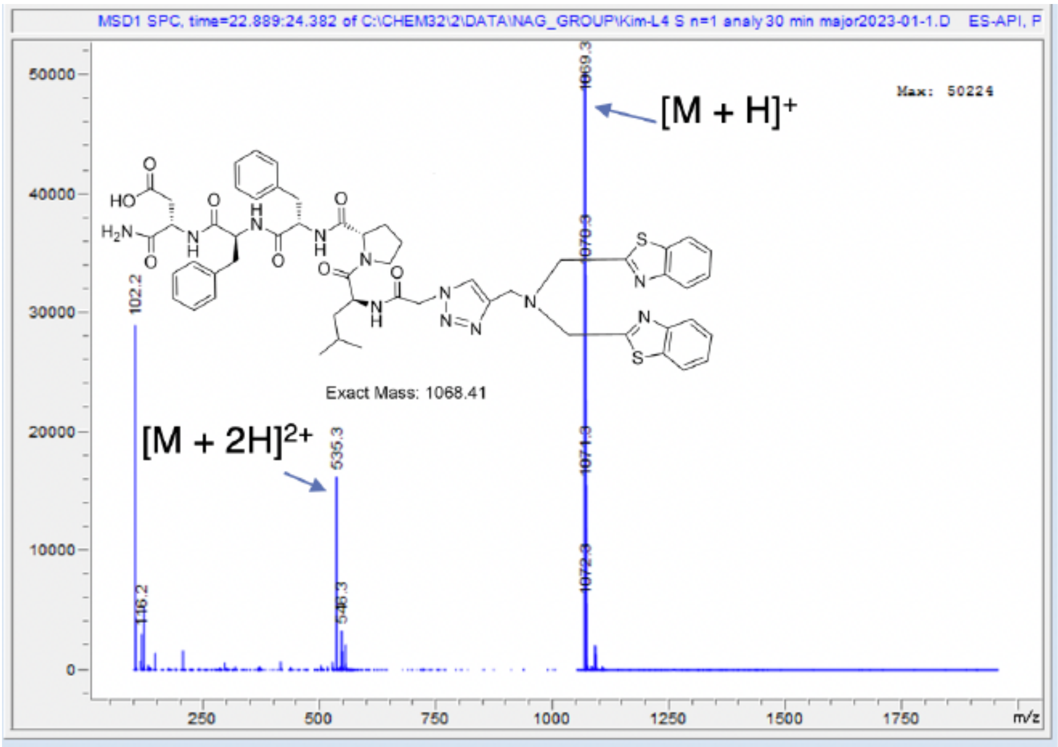

Figure S- 9: Synthesis of L1 on resin by amide coupling reaction

Side chain-protected peptide LPFFD on Rink amide resin was conjugated with the chelator A1 in anhydrous DMF and 2,6-lutidine for 48 hours at 45°C to form bifunctional ligand L1 on resin.

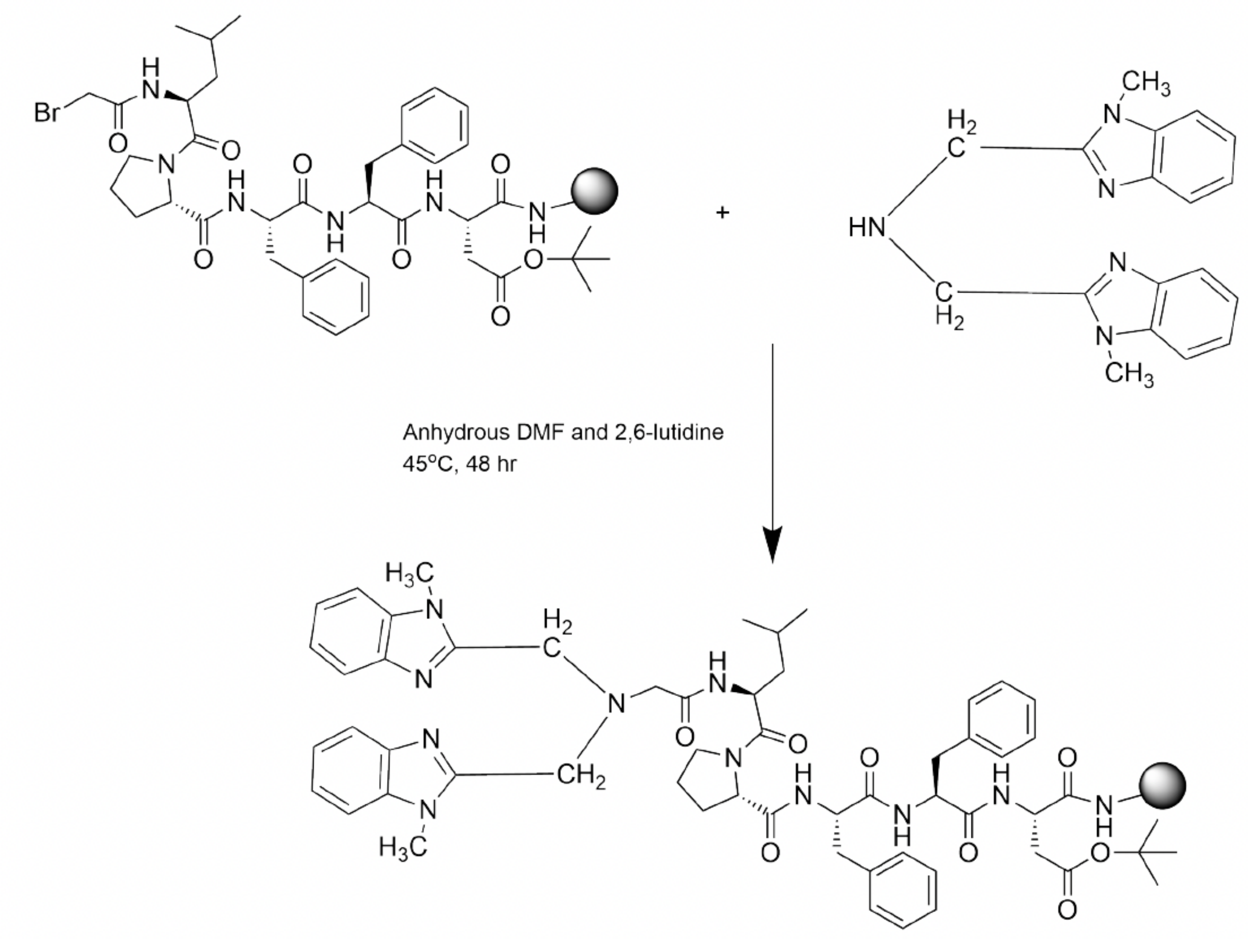
Anhydrous DMF and 2,6-lutidine
45°C, 48 hr

Figure S- 10: cleavage of bifunctional ligand L1 from resin

Bifunctional ligand L1 can be cleaved from Rink Amide resin to release L1 into solution by using 95% TFA, 2.5% TES, and 2.5% H_2O. The mixture was stirred at room temperature for 2 hours. The cleaved peptide was precipitated into cold diethyl ether, centrifugated to form a pellet, and then dissolved in water and lyophilized.

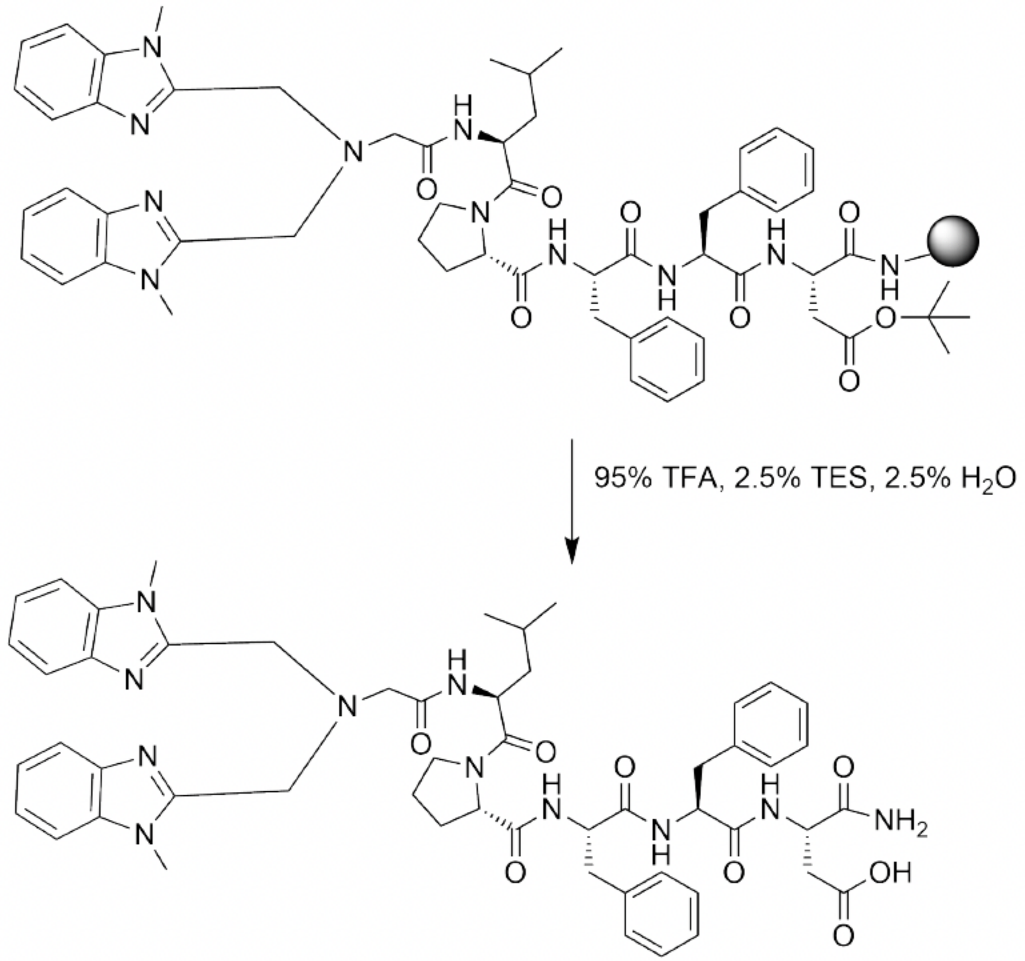

Figure S- 11: Synthesis of L2 on rink amide resin

The activation of LPFFD peptide by reaction with bromoacetic acid. The activated peptide was conjugated with A2 chelator in anhydrous DMF and 2,6-lutidine at 45°C for 48 hours.

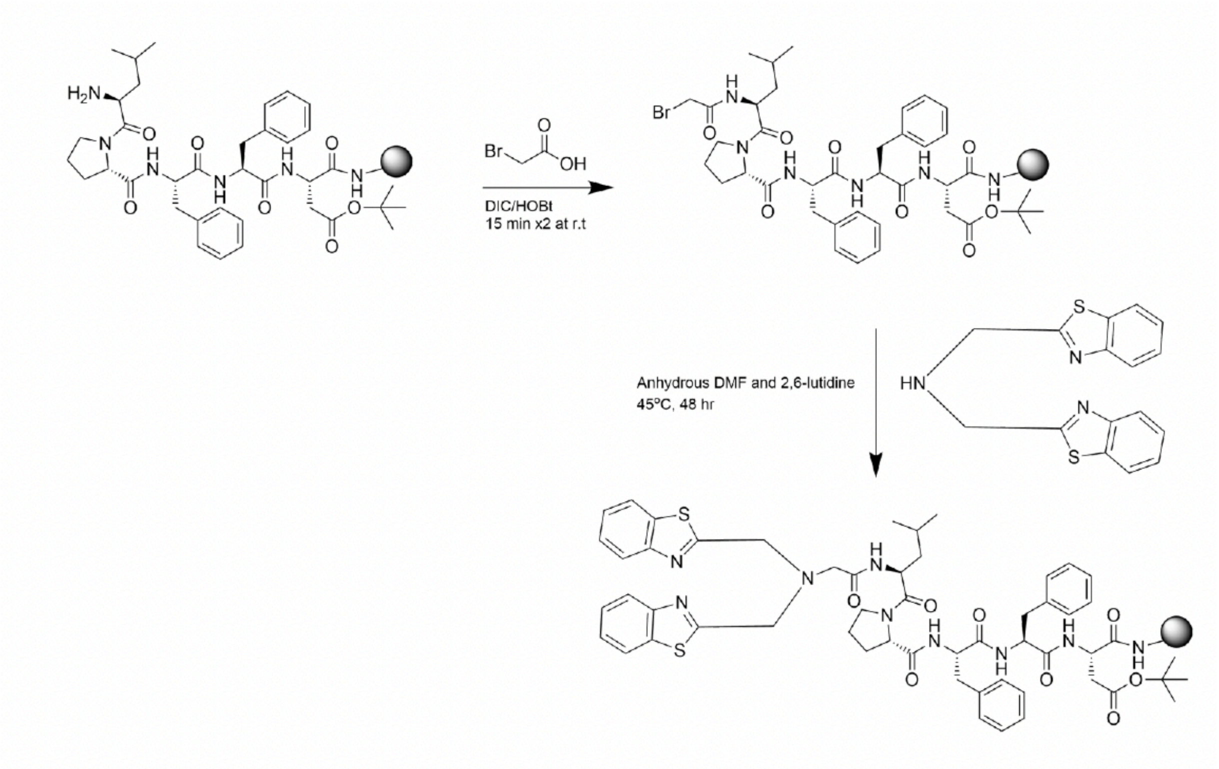

Figure S- 12: Cleavage of bifunctional ligand L2 from resin

Bifunctional ligand L2 was cleaved from Rink Amide resin using 95% TFA, 2.5% TES, and 2.5% H_2O. The mixture was stirred at room temperature for 2 hours. The cleaved peptide was precipitated into cold diethyl ether, centrifugated to form a pellet, and then dissolved in water and lyophilized.

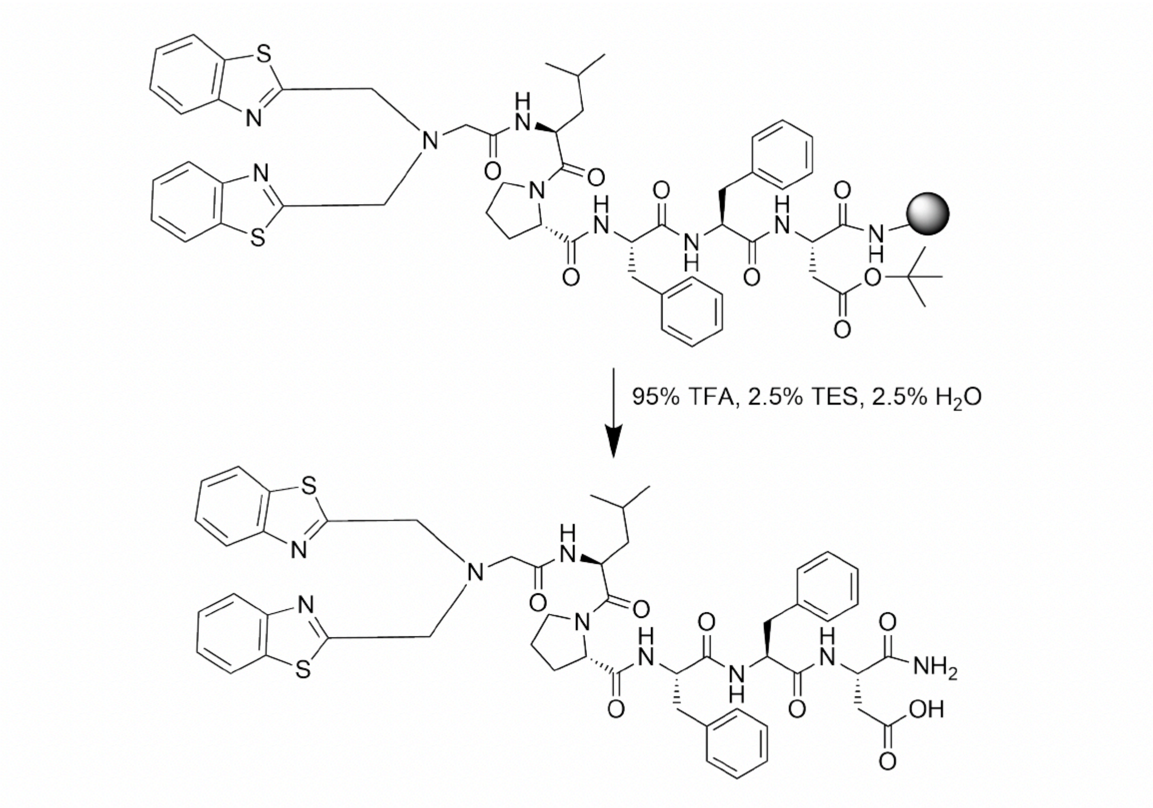

Figure S- 13: Synthesis of L3 on rink amide resin

A. The peptide LPFFD on the resin was coupled with 3 equivalents azidoacetic acid, 3 equivalents HATU, and 9 equivalents DIEA in NMP at room temperature overnight to add the azide group to the peptide. B. Chelator A2 was treated with propargyl bromide in anhydrous DMF and 2,6-lutidine at 50ºC overnight to add the alkyne group to the chelator. C. The azide-alkyne click reaction between the azide-containing LPFFD peptide and the alkyne-containing chelator forms L3.

A.

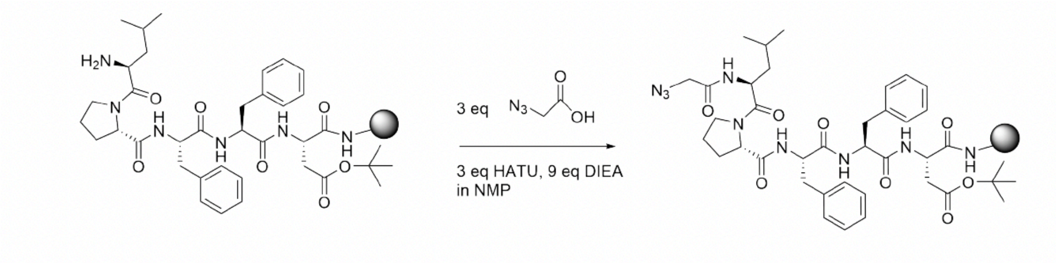

B.

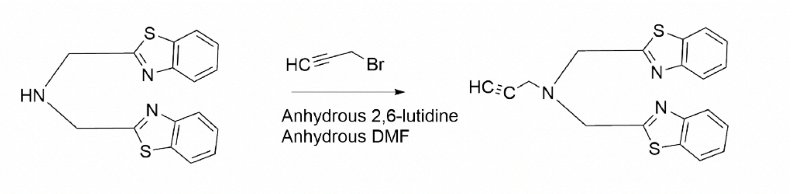

C.

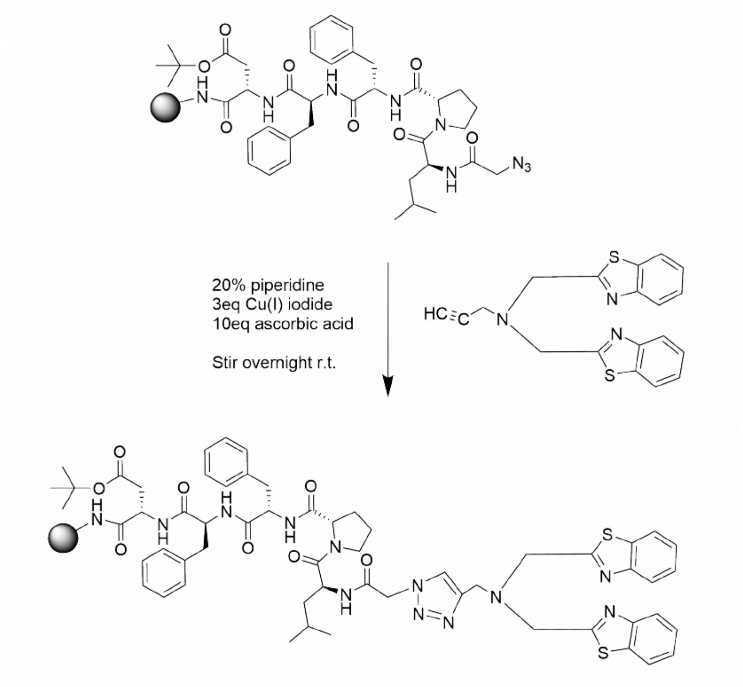

Figure S- 14: Cleavage of L3 from resin

Bifunctional ligand L3 on rink amide resin was cleaved to release L3 into solution by treatment with 95% TFA, 2.5% TES, and 2.5% H_2O. The mixture was stirred at room temperature for 2 hours. The cleaved peptide was precipitated into cold diethyl ether.

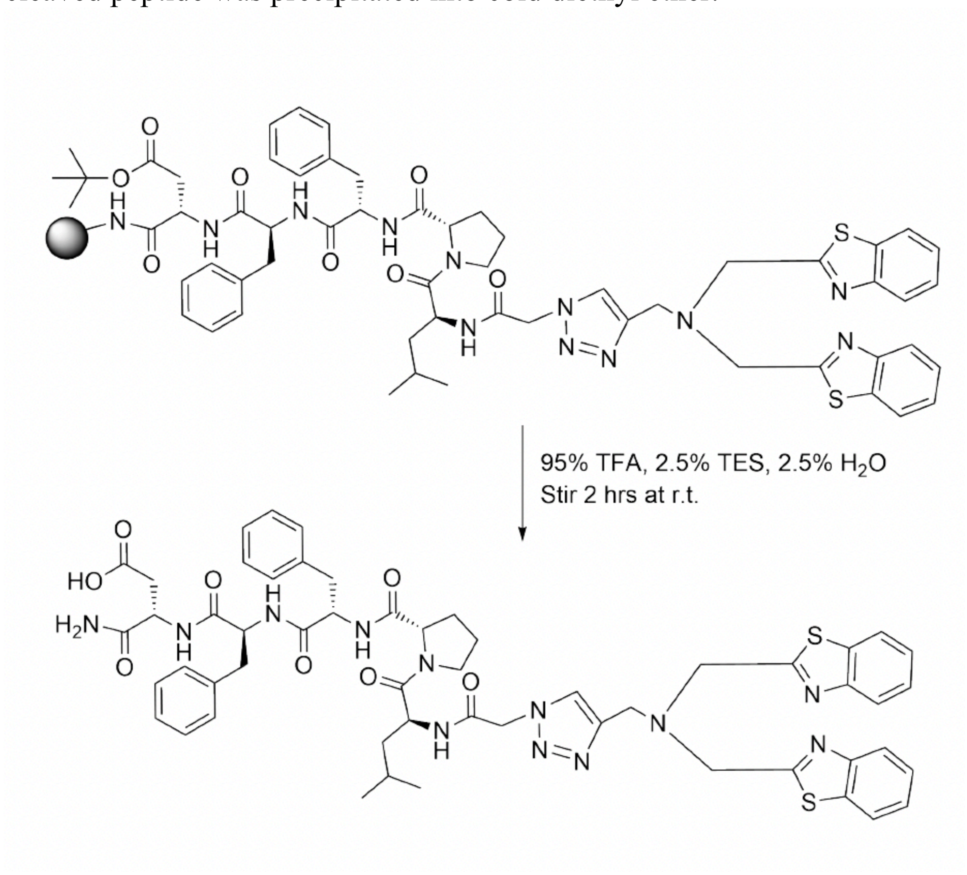

Figure S- 15: Absorbance spectra of L1, L2 and L3

A. Absorbance of 25 µM L1 and 10x dilution of L1 in 10 mM HEPES buffer pH 7.34. B. Absorbance of 6.5 µM L2 in 10 mM HEPES buffer pH 7.34. C. Absorbance of 17 µM L3 in 10 mM HEPES buffer pH 7.34.

A.

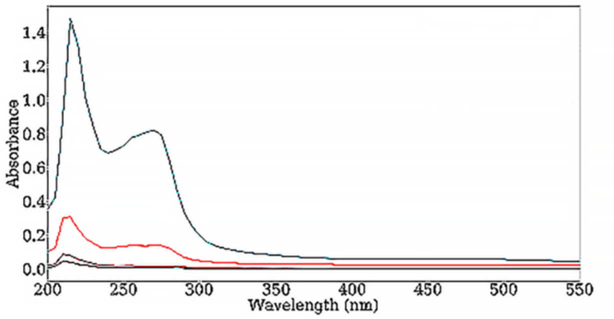

B.

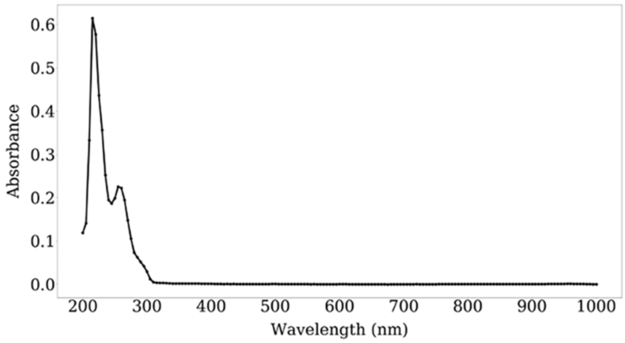

C.

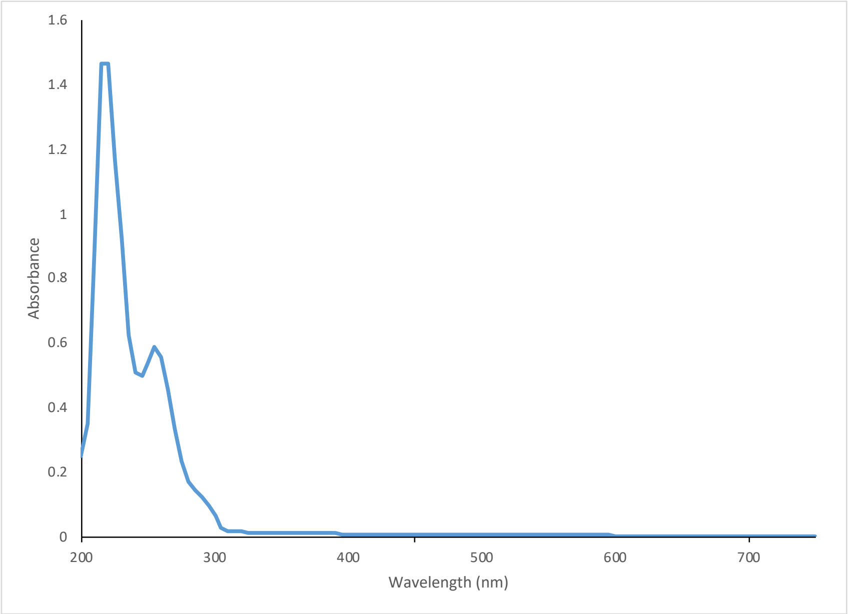

Figure S- 16: L3 solution fluorescence intensity constant over 24 hours.

A. Initial fluorescence at 0 hours

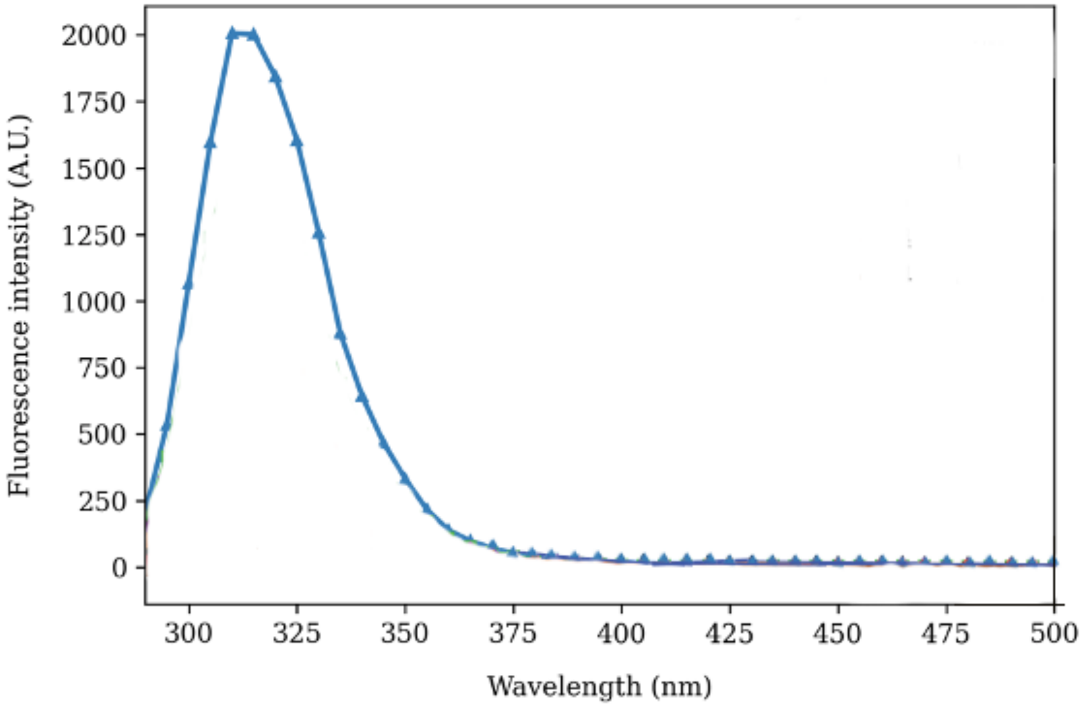

B. Fluorescence after 24 hours

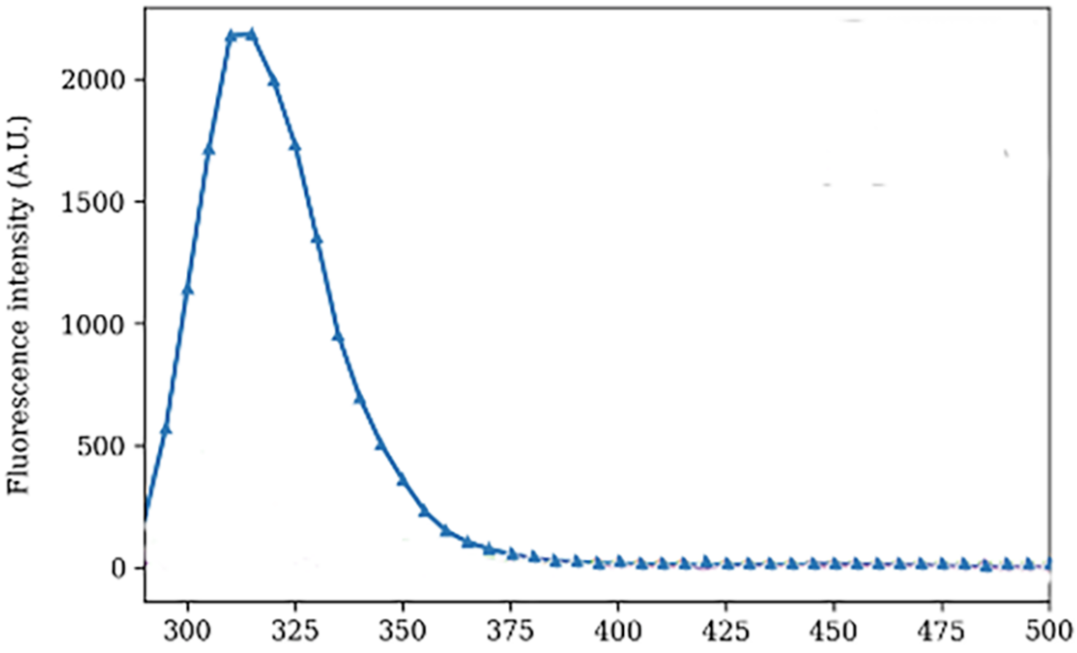

Figure S- 17: Selectivity comparison of 20 µM L1 for various metal ions.

The fluorescence spectrum of 20 µM L1 with different metal ions was measured from 290 to 500 nm. 20 µM L1 control had an intensity at around 1600 A.U (black). Adding Cu^{2+} decreased the intensity to around 400 A.U (blue). A lower fluorescence decrease was observed with the addition of Co^{2+} and Ni^{2+}. The addition of other metal ions did not lead to any significant change.

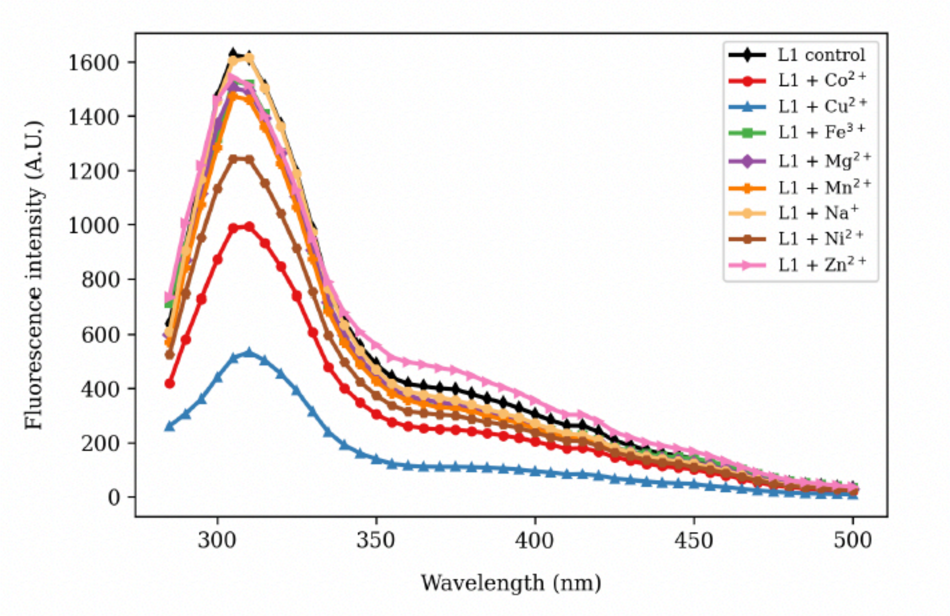

Figure S- 18: Normalized fluorescence of L1 on adding different metal ions at a 1:1 ratio.

The maximum change in fluorescence at 310 nm was normalized to one, and other changes were quantified as fractions.

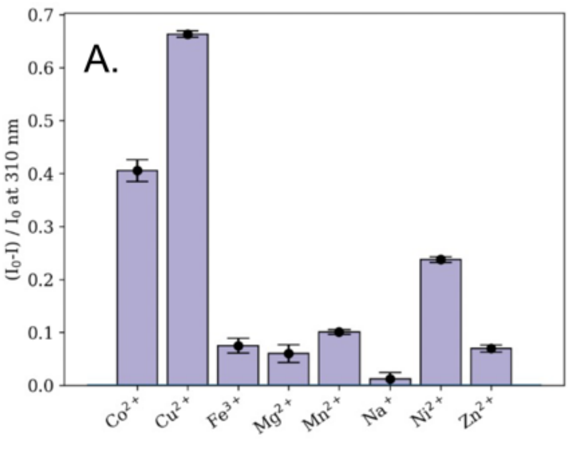

Figure S- 19: Selectivity comparison of 20 μM L2 for various metal ions.

The fluorescence spectrum of L2 with different metal ions was measured from 290 to 500 nm. 20 μM L2 had an intensity at around 400 A.U (black). Adding 20 μM Cu^{2+} decreased the intensity to around 300 A.U (blue line). The addition of other metal ions did not lead to any significant change.

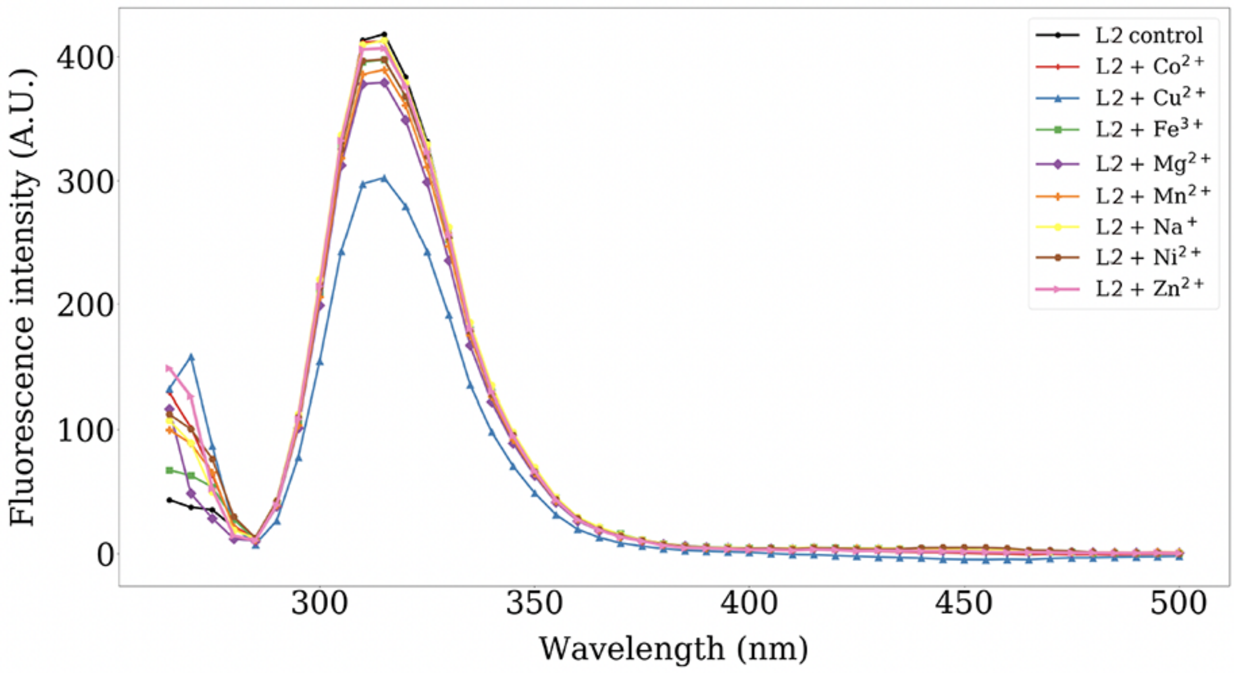

Figure S- 20: Selectivity comparison of 8 μM L2 for various metal ions

The fluorescence spectrum of 8 μM L2 with different metal ions measured from 290 to 500 nm. L2 control had an intensity at around 800 A.U (black). Adding different metal ions led to changes in fluorescence intensities.

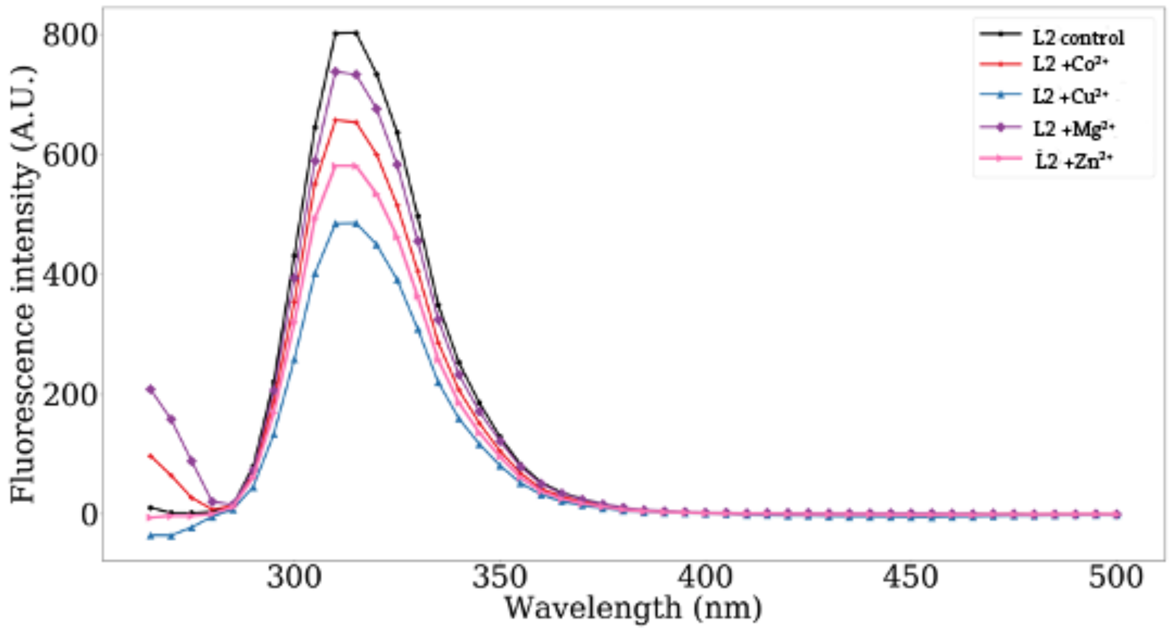

Figure S- 21: Selectivity comparison of 0.2 µM L2 for various metal ions

The fluorescence spectrum of 0.2 µM L2 with different metal ions measured from 290 to 500 nm. L2 control had an intensity at around 600 A.U (black). Adding different metal ions led to changes in fluorescence intensities, and the values decreased to around 500 A.U.

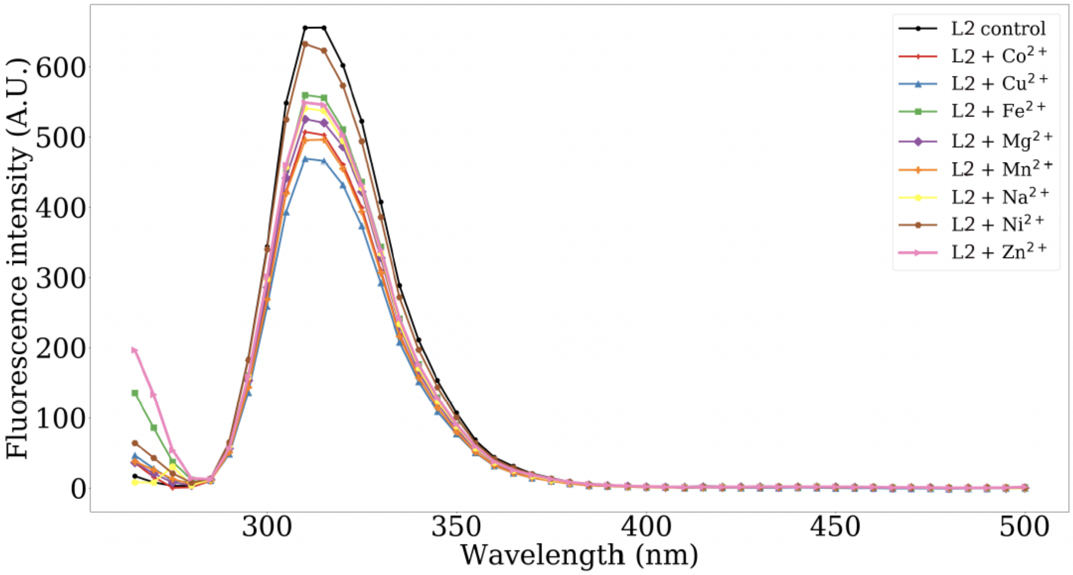

Figure S- 22: Variation of fluorescence intensity of L3 with pH change.

The fluorescence intensity of L3 in 10 mM HEPES buffer at different pH values was measured. Fluorescence intensities of 20 µM L3 differed at pH values from 6.0 to 8.0, and higher fluorescence intensities were observed at pH values of 7-8. The highest fluorescence was seen at pH 8.0, but high fluorescence was also seen at pH 7.5, close to the pH of 7.34 used in the experiments.

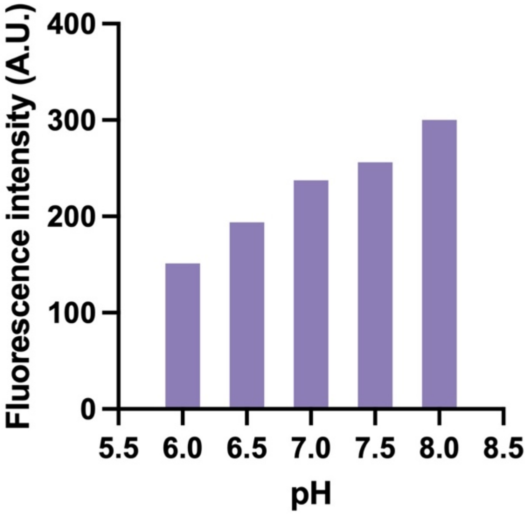

Figure S- 23: Selectivity comparison of 20 μM L3 for various metal ions

The fluorescence spectrum of 20 μM L3 with different metal ions measured from 290 to 500 nm. 20 μM L3 had an intensity at around 300 A.U (black). Adding Cu^{2+} decreased the intensity to around 70 A.U. (blue). The addition of other metal ions did not lead to any significant change.

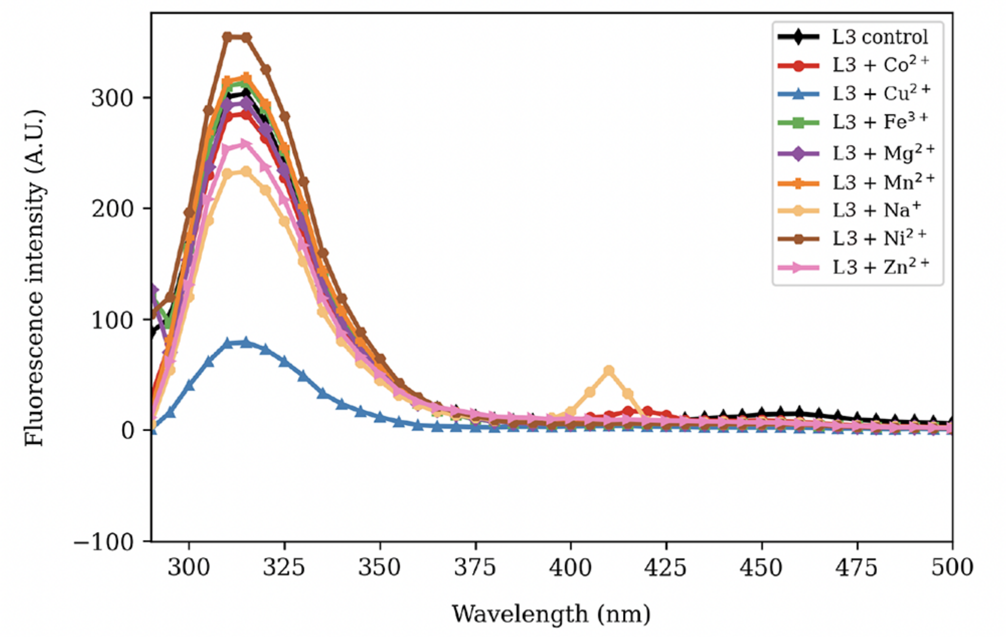

Figure S- 24: Variation of fluorescence intensity with concentration of L3

The fluorescence intensity of different concentrations of L3 in 10 mM HEPES buffer pH 7.34 was measured. The 20 μM solution was self-quenched and had lower fluorescence, while the 10x diluted solution of L3 (2 μM) had high fluorescence. Various dilutions in the 2 μM range were explored to determine the concentration that would work best for the later titration assay with metal ions.

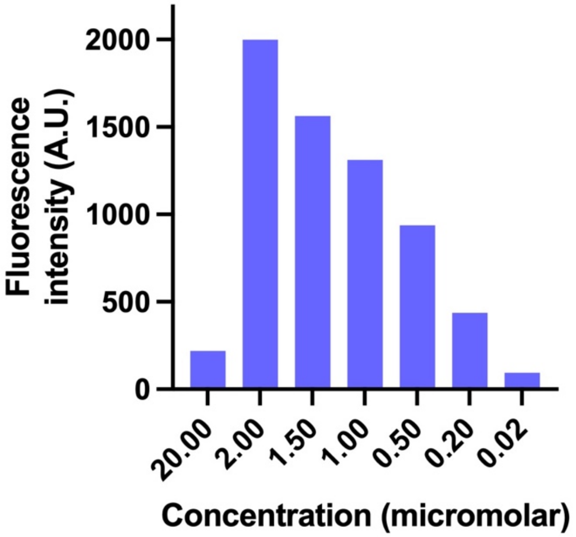

Figure S- 25: Ab42 tyrosine fluorescence at 310 nm in the absence of and presence of Cu^{2+}.

Fluorescence intensity at 310 nm of 20 µM Ab42 (black) excited at 280 nm decreased from 800 A.U. to 0 A.U. after incubation with 20 µM Cu^{2+} for 48 hours (red).

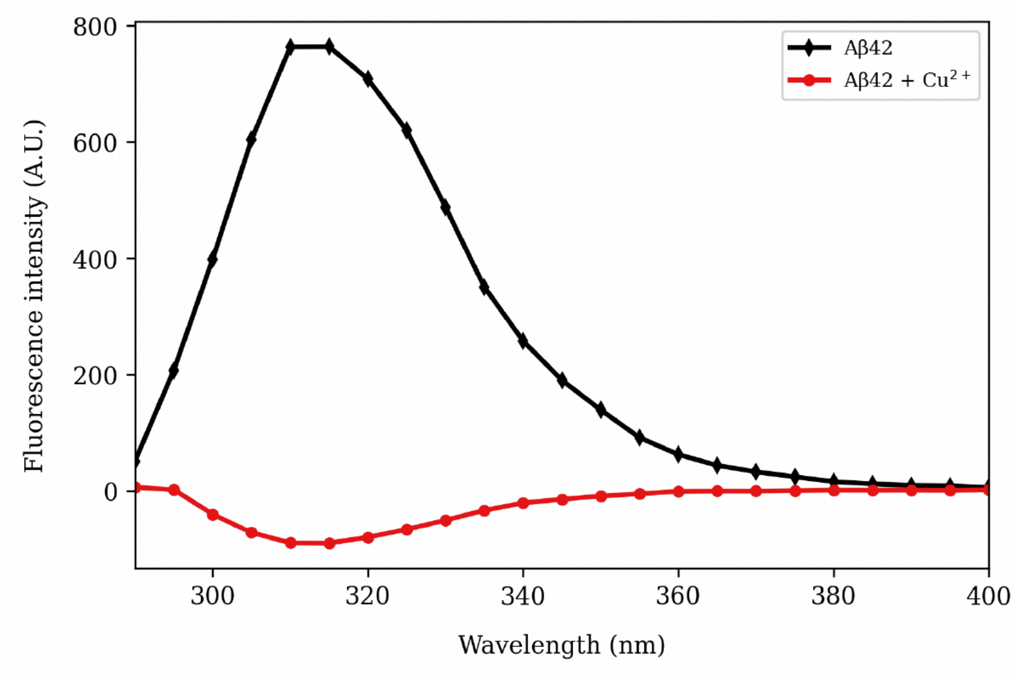

Figure S- 26: ThT fluorescence assay of 10 µM Ab42 in the presence of 10 µM Cu^{2+} and 10 µM L3

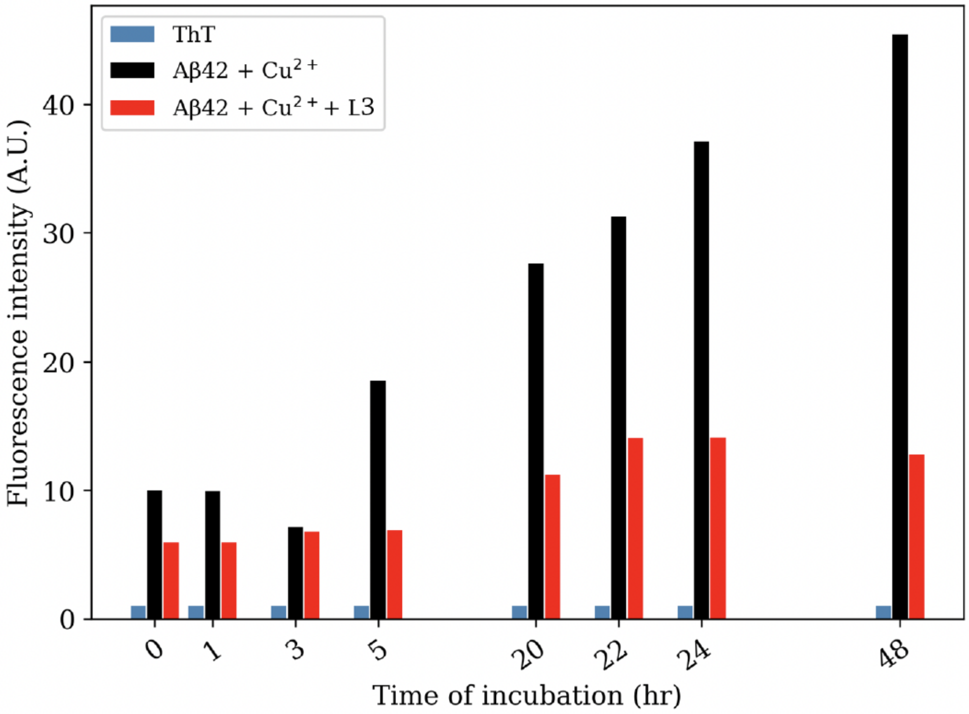

Figure S- 27: ThT fluorescence assay of 20 μM Ab42 and 20 μM L3 in absence of Cu^{2+}

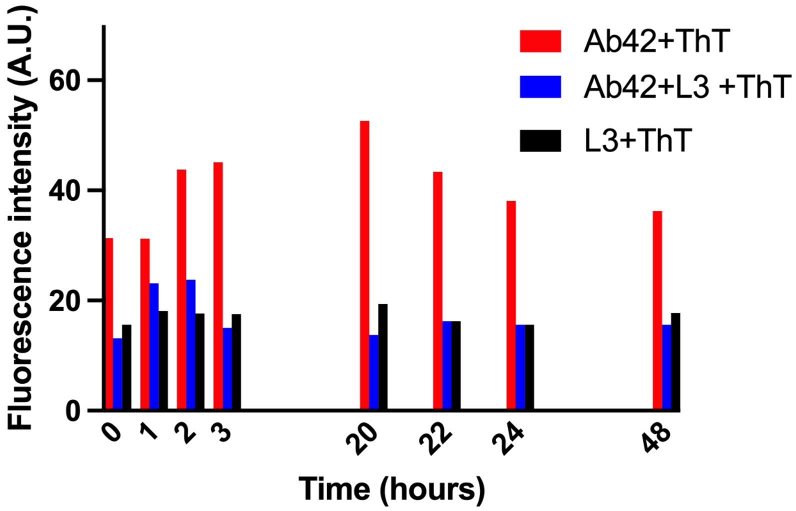

Figure S- 28: ThT fluorescence assay of 20 μM Ab42 and 20 μM Cu^{2+} in the presence of 10 μM and 20 μM L3

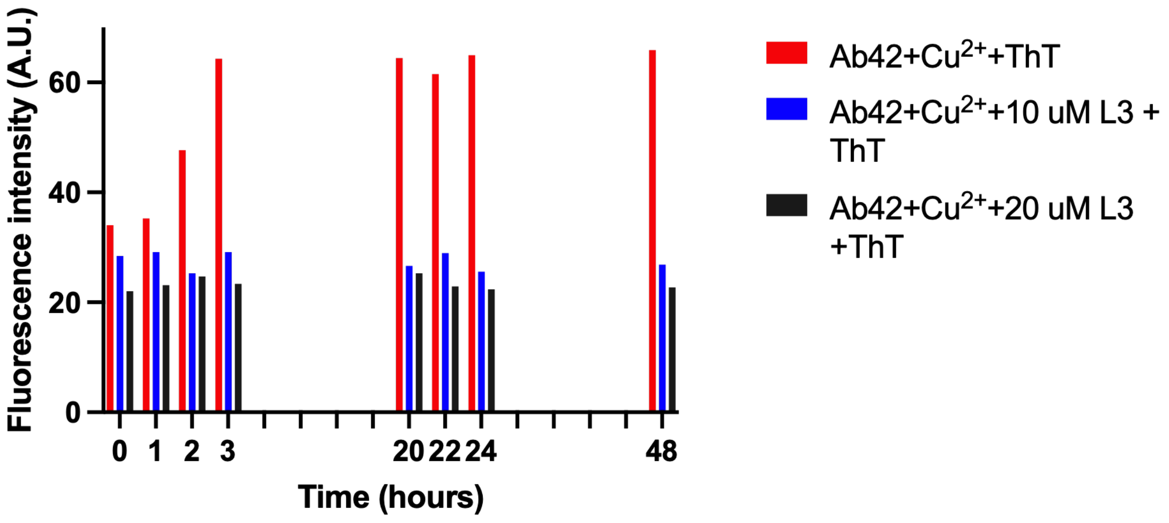

Figure S- 29: Bright field image of Ab42 and Ab42 and L3

A. 20 µM Ab42 was incubated with light shaking for 22 hours at 37°C in 10 mM HEPES buffer pH 7.34, and then its DIC image was taken. A few small aggregates are seen, and no large non-amyloidogenic aggregation was observed, indicating Cu^{2+} played a vital role in forming large non-amyloidogenic aggregates. B. 20 µM Ab42 was incubated with 20 µM L3 with light shaking for 22 hours at 37°C in 10 mM HEPES buffer pH 7.34, and then its DIC image was taken. The image showed many small particles formed with an average area of 2.1 μm^2. No significant change was observed in the images on adding L3 to Ab42 without copper.

A.

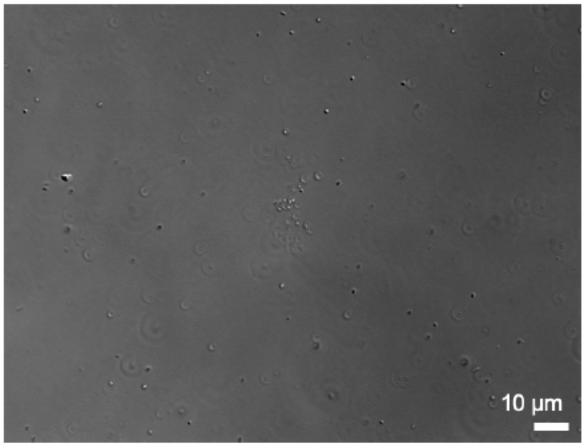

B.

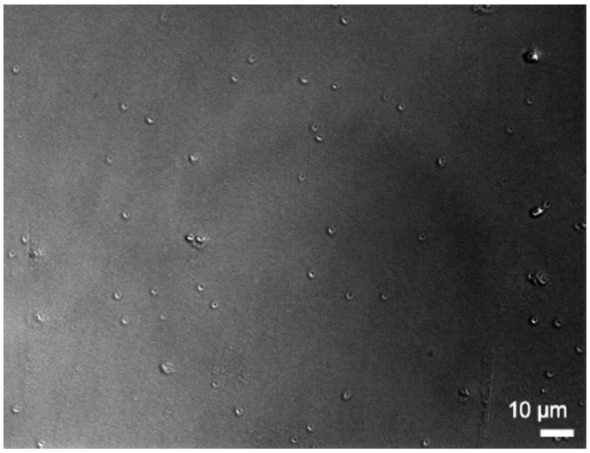

Supplement: Supplementary file 1 [file biosensors-14-00247-s001.zip › biosensors-2983391-supplementary.pdf]
